# Structural and Molecular Insight into Piperazine and Piperidine Derivatives as Histamine H_3_ and Sigma-1 Receptor Antagonists with Promising Antinociceptive Properties

**DOI:** 10.1021/acschemneuro.1c00435

**Published:** 2021-12-15

**Authors:** Katarzyna Szczepańska, Sabina Podlewska, Maria Dichiara, Davide Gentile, Vincenzo Patamia, Niklas Rosier, Denise Mönnich, Ma Carmen Ruiz Cantero, Tadeusz Karcz, Dorota Łażewska, Agata Siwek, Steffen Pockes, Enrique J. Cobos, Agostino Marrazzo, Holger Stark, Antonio Rescifina, Andrzej J. Bojarski, Emanuele Amata, Katarzyna Kieć-Kononowicz

**Affiliations:** † Department of Technology and Biotechnology of Drugs, Faculty of Pharmacy, Jagiellonian University Medical College, Medyczna 9, Kraków 30-688, Poland; § Maj Institute of Pharmacology, Polish Academy of Sciences, Smętna 12, Kraków 31-343, Poland; ⊥ Department of Drug and Health Sciences, University of Catania, V.le A. Doria, 95125 Catania, Italy; ∥ Institute of Pharmacy, Faculty of Chemistry and Pharmacy, University of Regensburg, Universitätsstraße 31, D-93053 Regensburg, Germany; ∇ Department of Pharmacology and Neurosciences Institute (Biomedical Research Center) and Biosanitary Research Institute ibs.GRANADA, University of Granada, Avenida de la Investigación 11, 18016 Granada, Spain; ◆ Department of Pharmacobiology, Faculty of Pharmacy, Jagiellonian University Medical College, Medyczna 9, Kraków 30-688, Poland; ○ Institute of Pharmaceutical and Medicinal Chemistry, Heinrich Heine University Düsseldorf, Universitaetsstr. 1, 40225 Duesseldorf, Germany

**Keywords:** histamine H_3_ receptor, sigma-1 receptor, sigma-2 receptor, piperazine derivatives, piperidine derivatives, dual targeting compounds, molecular docking, dynamics, functional characterization

## Abstract

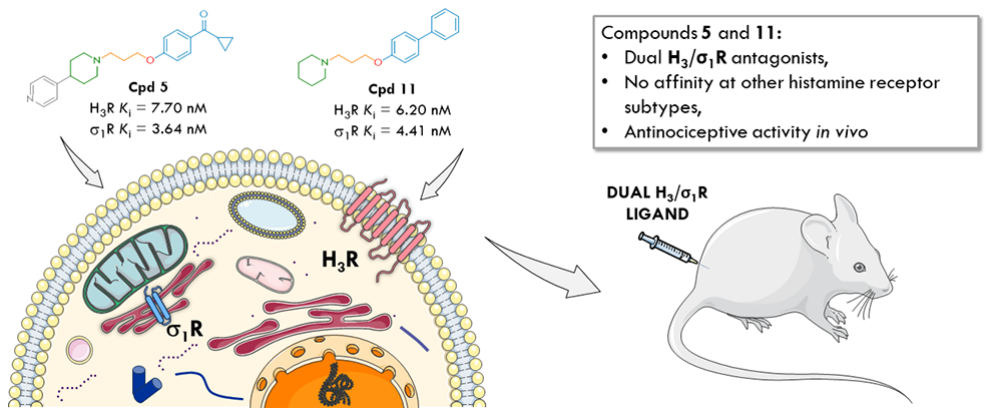

In an attempt to extend recent studies showing that some clinically evaluated histamine H_3_ receptor (H_3_R) antagonists possess nanomolar affinity at sigma-1 receptors (σ_1_R), we selected 20 representative structures among our previously reported H_3_R ligands to investigate their affinity at σRs. Most of the tested compounds interact with both sigma receptors to different degrees. However, only six of them showed higher affinity toward σ_1_R than σ_2_R with the highest binding preference to σ_1_R for compounds **5**, **11**, and **12**. Moreover, all these ligands share a common structural feature: the piperidine moiety as the fundamental part of the molecule. It is most likely a critical structural element for dual H_3_/σ_1_ receptor activity as can be seen by comparing the data for compounds **4** and **5** (hH_3_R *K*_i_ = 3.17 and 7.70 nM, σ_1_R *K*_i_ = 1531 and 3.64 nM, respectively), where piperidine is replaced by piperazine. We identified the putative protein–ligand interactions responsible for their high affinity using molecular modeling techniques and selected compounds **5** and **11** as lead structures for further evaluation. Interestingly, both ligands turned out to be high-affinity histamine H_3_ and σ_1_ receptor antagonists with negligible affinity at the other histamine receptor subtypes and promising antinociceptive activity *in vivo*. Considering that many literature data clearly indicate high preclinical efficacy of individual selective σ_1_ or H_3_R ligands in various pain models, our research might be a breakthrough in the search for novel, dual-acting compounds that can improve existing pain therapies. Determining whether such ligands are more effective than single-selective drugs will be the subject of our future studies.

## 1 Introduction

In recent decades, significant research efforts have been invested in discovering and developing therapeutics that modulate individual disease-modifying targets. Although this approach has led to growth in the industry and numerous successful drugs reaching the market, only a few new drugs act at novel molecular targets. The limitations of many monotherapies can be overcome by attacking the disease system on multiple fronts.^1^ Multitarget therapeutics may be more effective and less vulnerable to adaptive resistance because the biological system is less able to compensate for the effects of two or more drugs simultaneously.^2^ Indeed, multicomponent drugs are now standard in therapeutic areas such as cancer, diabetes, and psychiatric or degenerative central nervous system (CNS) disorders, paradoxically composed of agents initially developed as single-target drugs.^3,4^

**Figure 1:**
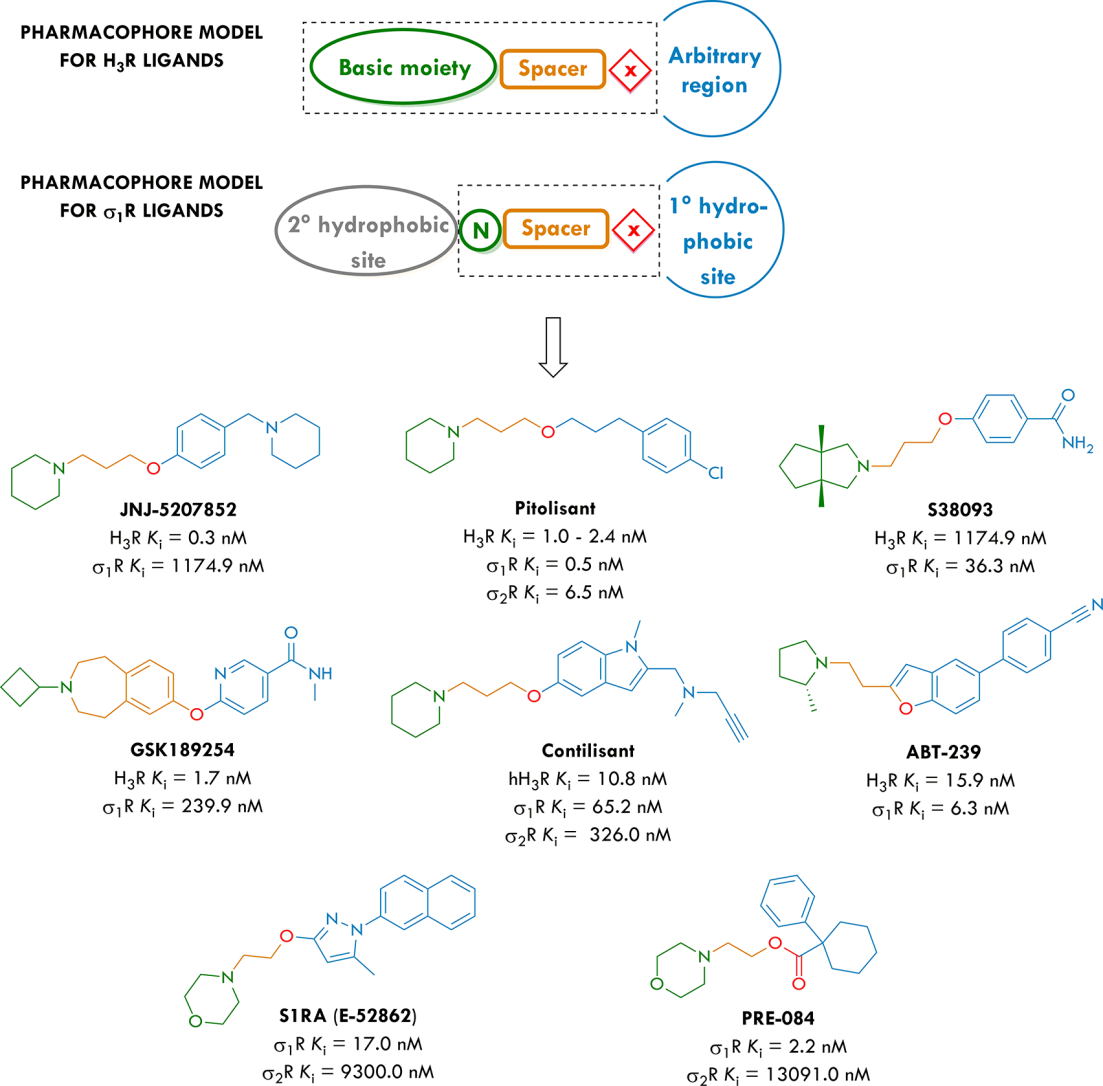
General pharmacophore models for H_3_ and σ_1_ receptor ligands (N, nitrogen atom, X, single heteroatom or group with heteroatom), histamine H_3_ and σ_1_ affinity values of clinically evaluated H_3_R antagonists,^17^ and structures of reference σ_1_R ligands, S1RA (σ_1_R antagonist) and PRE-084 (σ_1_R agonist).

Histamine H_3_ receptors (H_3_Rs) belong to the family of G-protein-coupled receptors (GPCRs) and provide a broad spectrum of neuromodulatory functions in the CNS.^5^ They have been described as both presynaptic autoreceptors regulating the synthesis and release of histamine and heteroreceptors modulating the release of neurotransmitters such as acetylcholine, dopamine, norepinephrine, serotonin, γ-aminobutyric acid, glutamate, and substance P.^6,7^ Pharmacological data reveal potentially beneficial outcomes of H_3_R antagonists or inverse agonists for the treatment of schizophrenia, Alzheimer’s and Parkinson’s diseases, obesity, narcolepsy, and attention-deficit hyperactivity disorder (ADHD),^8^ also as multitargeting ligands.^9−11^ With the recent market approval of pitolisant (Wakix), the interest in clinical applications of novel multifunctional H_3_R antagonists has clearly increased.^12−16^ Interestingly, the latest studies have shown that some clinically evaluated H_3_R receptor antagonists possess nanomolar affinity at sigma-1 receptor (σ_1_R) binding sites, suggesting that this feature might play an essential role in their overall efficacy (Figure 1).^17−19^ This discovery may be a breakthrough in the therapeutic use of these compounds and opens a brand-new research area in the search for novel drugs.^20^

Sigma receptors (σRs), initially recognized as one of the opioid receptor subtypes based on binding with benzomorphan compounds^21^ are now considered a distinct class of proteins divided into two subtypes, the sigma-1 receptor (σ_1_R) and the sigma-2 receptor^22^ (σ_2_R). The σ_1_R has been identified as a chaperone protein that can interact with various receptors and channels, acting as a regulatory subunit.^23,24^ Consequently, the σ_1_R regulates several neurotransmitter systems, including the glutamatergic, dopaminergic, serotonergic, noradrenergic, and cholinergic systems.^25−27^ Thus, σ_1_R ligands represent potential therapeutic agents for treating several neuropsychiatric and neurodegenerative disorders, drug abuse, and pain (among other possible therapeutic indications).^28−33^ In this context, the highly selective σ_1_ antagonist S1RA is in phase II clinical trials for pain treatment, with an intended indication for enhancing opioid analgesia and ameliorating neuropathic pain.^34^ On the other hand, pitolisant binds to human σ_1_R with a sub-nanomolar *K*_i_ value of 0.5 nM and shows a functional agonism of σ_1_ receptor-mediated calcium flux with an EC_50_ of 402 nM.^35^ Regarding σ_2_R, it binds with a *K*_i_ of 6.5 nM and an IC_50_ of 8.55 nM. In a σ_2_ receptor-mediated calcium flux functional assay, pitolisant did not elicit agonist activity but behaved as an antagonist as it decreased haloperidol-induced calcium release with an IC_50_ of 10 μM.^35^

Despite the high diversity of H_3_R antagonists, these structures share a similar design pattern. The pharmacophore (Figure 1) contains a basic tertiary amine, a linker (commonly a linear propyloxy or structurally constrained chain), a central core, and an arbitrary region with high diversity, such as second basic, acidic, lipophilic, or polar moieties of different sizes.^36,37^ For σ_1_R ligands, numerous structure–activity relationship (SAR) studies have been performed in an attempt to develop a common pharmacophore model.^38−41^ Generally, it consists of three main sites: a central amine site that includes an essential proton acceptor site flanked by two hydrophobic domains, a primary hydrophobic site that binds phenyl group “B”, and a secondary binding site that binds phenyl group “A” (Figure 1, gray for B, blue for A). On the other hand, σ_2_R ligands also consist of an amine binding site flanked by two hydrophobic sites; in fact, there is a striking similarity to the σ_1_R binding requirements (and indeed most compounds described in the literature bind to both σ_1_ and σ_2_ receptors).^42^ Most importantly, in the case of both pharmacophore models for histamine H_3_ and σ_1_R ligands (Figure 1), some common structural elements are noticeable, namely, a basic moiety connected via a heteroatomic linker that is directly attached to the arbitrary region. Furthermore, such a structural configuration of these elements is an example of merged pharmacophores, the most promising strategy in the search for novel multicomponent drugs.^43,44^

Considering the above, we selected representative structures **1**–**20** (Table 1) among our previously reported H_3_R ligands to investigate their affinity at σ_1_R and σ_2_R, as we wondered if their reported high preclinical efficacy *in vivo* might be related to a synergistic effect of dual H_3_R and σ_1_R modulation.^45−48^ Also, for the most promising dual-acting compounds, we determined their agonistic and antagonistic activities toward the H_3_R and σ_1_R, as those parameters are crucial for further *in vivo* studies. Finally, using *in silico* pharmacophore modeling and docking algorithms, we demonstrated probable protein–ligand interactions responsible for the compounds’ activity.

**Table 1:**
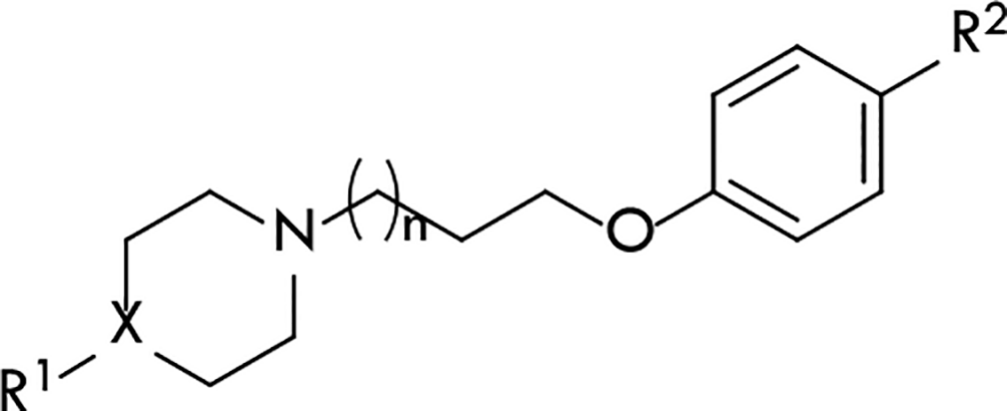
Structures of Compounds **1**–**20** and Their *In Vitro* Binding Affinities at the Human Histamine H_3_ receptor (hH_3_R) and Rat Sigma-1 (σ_1_R) and Sigma-2 (σ_2_R) Receptors

					*x̅* [CI 95%]^a^			
compd	*n*	X	R^1^	R^2^	hH_3_R *K*_i_ [nM]	σ_1_R *K*_i_ [nM]	σ_2_R *K*_i_ [nM]	σ_2_/σ_1_ratio
1	1	N	pyridin-4-yl	ethyl	40.4 [17.1, 95.9]^b^	592 [281, 1246]	64.3 [22.3, 185]	0.1
2	1	N	pyridin-4-yl	*tert*-butyl	16.0 [8.1, 31.7]^c^	112 [77, 164]	130 [97.8, 172]	1.2
3	1	N	pyridin-4-yl	acetyl	10.2 [3.6, 29.0]^d^	1409 [480, 4137]	247 [117, 522]	0.2
4	1	N	pyridin-4-yl	cyclopropylmethanone	3.17 [2.56, 3.91]^e^	1531 [652, 3593]	101 [49.3, 205]	0.1
5	1	CH	pyridin-4-yl	cyclopropylmethanone	7.70 [3.62, 16.38]^e^	3.64 [1.81, 7.30]	22.4 [9.36, 53.8]	6.2
6	1	N	pyridin-4-yl	phenyl	21.1 [3.8, 116]^b^	638 [260, 1566]	108 [46.7, 250]	0.2
7	1	N	pyridin-4-yl	4-cyanophenyl	7.86 [2.82, 21.90]^e^	2958 [629, 13904]	75.2 [33.1, 171]	<0.1
8	1	N	pyridin-4-yl	benzoyl	3.12 [0.66, 14.60]^b^	726 [219, 2413]	29.2 [22.2, 38.5]	<0.1
9	1	N	pyridin-4-yl	4-chlorobenzoyl	23.0 [12.4, 42.4]^e^	641 [340, 1209]	32.4 [19.9, 52.6]	0.1
10	1	N	pyridin-4-yl	4-fluorobenzoyl	5.84 [3.35, 10.19]^e^	1309 [373, 4599]	164 [59.2, 454]	0.1
11	1	CH	H	phenyl	6.20 [1.90, 20.40]^f^	4.41 [2.62, 7.40]	67.9 [41.0, 112]	15.4
12	1	CH	H	benzoyl	22.0 [6.0, 83.0]^f^	14.8 [8.28, 26.3]	96.2 [47.1, 196]	6.5
13	2	N	pyridin-4-yl	*tert*-butyl	37.8 [24.0, 59.4]^c^	51.8 [22.5, 119]	175 [67.0, 459]	3.4
14	2	N	pyridin-4-yl	*tert*-pentyl	120 [63, 230]^c^	285 [123, 659]	101 [40.5, 251]	0.4
15	2	N	pyridin-4-yl	acetyl	115 [26.8, 493]^d^	>10 000	1795 [579, 5564]	<0.2
16	4	N	pyridin-4-yl	acetyl	12.7 [4.4, 36.9]^b^	37.8 [20.9, 69.6]	151 [65.9, 345]	4.0
17	4	N	pyridin-4-yl	propionyl	16.9 [8.0, 36.0]^b^	248 [140, 439]	110 [56.4, 215]	0.4
18	4	N	pyridin-4-yl	*tert*-butyl	397 [220, 715]^b^	255 [104, 626]	179 [87.9, 363]	0.7
19	6	N	pyridin-4-yl	acetyl	40.5 [12.3, 134]^b^	408 [104, 1598]	59.7 [24.3, 147]	0.1
20	6	N	pyridin-4-yl	propionyl	38.9 [9.5, 159]^b^	274 [138, 544]	65.9 [30.1, 144]	0.2
S1RA					>10000^f^	17.0^g^	9300^g^	547.1
PRE084					>10000^f^	2.2^g^	>10000^g^	>4500
RHM-4^h^						2150^i^	0.26^i^	0.00012
PIT^j^					1.0–2.4^k^	0.5^k^	6.5^k^	13

a Given data represent mean values within the 95% confidence interval (CI).

b Data published in ref (47).

c Data published in ref (45).

d Data published in ref (46).

e Data published in ref (48).

f Data not published yet.

g Data published in ref (20).

h *N*-(4-(6,7-Dimethoxy-3,4-dihydroisoquinolin-2(1*H*)-yl)butyl)-5-iodo-3-methoxy-2-(methylperoxy)benzamide.

i Data published in ref (22).

j Pitolisant.

k Data published in ref (35).

## 2 Results and Discussion

### 2.1 Pharmacology

#### 2.1.1 Affinity at σRs and H_3_R

*In vitro* affinity data are assembled in Table 1. First of all, almost all compounds (except **15**) showed more or less significant affinity toward both sigma receptors with different binding affinities. However, only six showed higher affinity toward σ_1_R than σ_2_R with the highest binding preference to σ_1_R for compounds **11**, **12**, and **5**. Interestingly, all these ligands share a common structural feature: the piperidine moiety in their basic part. It is most likely a key structural element for dual H_3_/σ_1_ receptor affinities, as shown by comparing compounds **4** and **5** (hH_3_R *K*_i_ = 3.17 and 7.70 nM; σ_1_R *K*_i_ = 1531 and 3.64 nM, respectively), which differ only in the basic part. Moreover, replacing the piperazine ring with piperidine did not significantly affect the affinity at H_3_R, which can also be deduced by comparing data from compounds **4** and **5**. Furthermore, **11** showed the highest binding preference to σ_1_R among all tested compounds (hH_3_R *K*_i_ = 6.2 nM, σ_1_R *K*_i_ = 4.41 nM, and σ_2_R *K*_i_ = 67.9 nM). In the case of piperazine derivatives, which interact more strongly with the σ_1_R than the σ_2_R, there is no evident influence of the alkylic linker length on their affinity, which is shown with compounds **13** and **16** (hH_3_R *K*_i_ = 37.8 and 12.7 nM, σ_1_R *K*_i_ = 51.8 and 37.8 nM, respectively). This is mainly because these ligands have slightly different groups in the lipophilic part (*tert*-butyl and acetyl, respectively). For all the described piperazine derivatives, the effect of the alkyl chain can only be observed in regards to H_3_R, where the extension of the linker length decreased the affinity of *tert*-butyl analogues **2**, **13**, and **18** (hH_3_R *K*_i_ = 16.0, 37.8, and 397 nM, respectively).

Further studies are needed considering the influence of a distal regulatory region of the compounds on their affinity at the desired biological targets. All ligands with a selectivity index greater than 1 had structurally different moieties in this part; hence it can be concluded that *tert*-butyl, cyclopropylmethanone, phenyl, benzoyl, and acetyl groups were well tolerated in the σ_1_R binding pocket. Compound **15** turned out to be a selective H_3_R ligand with very low affinity at the σRs and, therefore, could be used as a reference ligand in further studies. Undoubtedly, the piperidine ring has been defined as the most influential structural element on compounds’ activity at the σ_1_R while maintaining the affinity toward the H_3_R and a moderate (but still acceptable) selectivity profile in terms of the σ_2_R. Therefore, **5** and **11** were selected as lead structures for further evaluation. Moreover, in this study, we have also tested for the first time the affinity at the H_3_R of reference σ_1_R ligands S1RA and PRE-084. The obtained results indicate their selectivity toward σ_1_R.

#### 2.1.2 Affinity at Other Histamine Receptors

To check the selectivity of our lead structures, radioligand binding studies at other histamine receptor subtypes were carried out. As the results for **5** were already presented in our previous work^48^ (hH_1_R *K*_i_ > 10 000 nM, hH_2_R *K*_i_ > 10 000 nM, hH_4_R *K*_i_ > 100 000 nM), compound **11** in its oxalate form was tested at human recombinant histamine H_1_, H_2_, and H_4_ receptor subtypes stably expressed in HEK293T cells. Obtained results clearly indicate high selectivity of the tested derivative toward human H_3_R (hH_1_R *K*_i_ > 10 000 nM, hH_2_R *K*_i_ > 100 000 nM, hH_4_R *K*_i_ > 10 000 nM).

#### 2.1.3 Intrinsic Activity toward H_3_R

**Figure 2:**
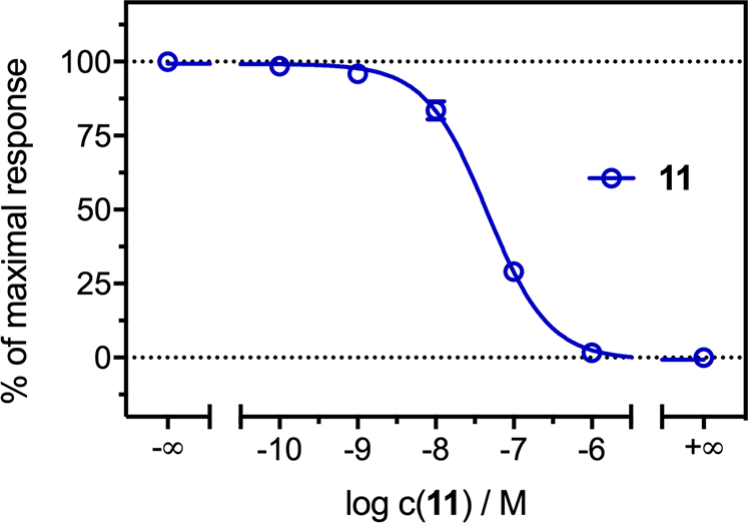
Concentration–response curve of compound **11** in the mini-G protein recruitment assay in HEK293T cells stably expressing the H_3_R-NlucC/NlucN-mGsi. Experiments were performed in the presence of histamine (*c* = 10 μM, antagonist mode). Data represent mean ± SEM from three independent experiments, each performed in triplicate.

To identify the lead compounds’ functional efficacy, their intrinsic activity was tested in the mini-G protein recruitment assay in response to H_3_R stimulation. The assay relies on the split-luciferase complementation technique^49^ and meets the demands of a sufficiently high dynamic range without radioactivity.^50^ Again, the antagonistic properties of **5** were previously described^48^ (hH_3_R *K*_b_ = 18.84 nM); therefore, this time, the intrinsic activity of **11** was tested (hH_3_R *K*_b_ = 11.38 nM). The concentration–response curve of compound **11** is presented in Figure 2.

#### 2.1.4 *In Vivo* Pharmacological Activity

**Figure 3:**
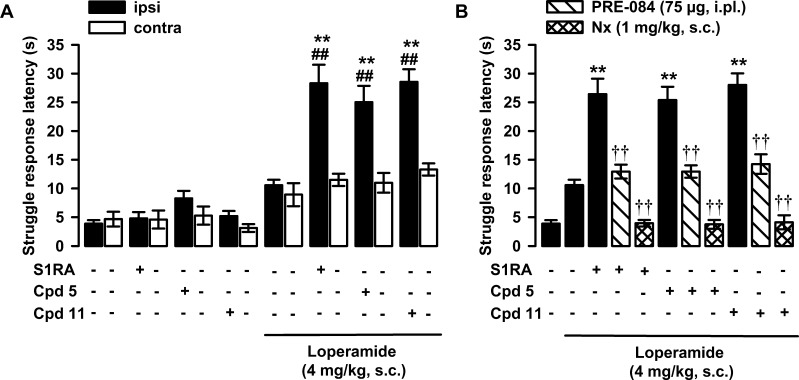
Effects of S1RA and compounds **5** and **11** on loperamide-induced antinociception. The results represent the struggle response latency during stimulation with 450 g pressure in mice intraplantarally (ipl) administered S1RA (100 μg), **5** (100 μg), **11** (50 μg), or saline and treated subcutaneously (sc) with loperamide (4 mg/kg) or its solvent (1% DMSO in ultrapure water). (A) Effect of treatments on the response latency to mechanical stimulation in the paw ipl injected with the σ_1_R ligands (ipsi) and in the contralateral paw (contra). (B) Effect of the ipl administration of PRE-084 (75 μg), and the sc administration of naloxone (Nx, 1 mg/kg) on the potentiation of loperamide-induced antinociception by S1RA, **5**, and **11**. Each bar and vertical line represents the mean ± SEM of values obtained in 6–8 animals. Two-way analysis of variance followed by the Bonferroni test was used to determine statistically significant differences between (A and B) the values obtained in the group treated with the solvent of the drugs and the rest of the groups (*P < 0.05, **P < 0.01), (A) between the ipsi and the contra paws (^##^*P* < 0.01) and (B) between the values of the ipsi paw from loperamide-treated mice injected with S1RA, **5**, or **11** alone or coadministered with PRE-084 or with the association with Nx (^††^*P* < 0.01).

As it is well-known that σ_1_R antagonism enhances opioid analgesia,^30,33,70^ we tested the effects of compounds **5** and **11** on antinociception induced by the opioid agonist loperamide. It is worth mentioning that loperamide is known to lack affinity for σ_1_R;^70^ S1RA was used as a σ_1_R reference antagonist. Both S1RA and **5** were administered intraplantarally (ipl) at a dose of 100 μg, while **11** (due to solubility problems) was tested at a dose of 50 μg. The antinociceptive effect of the treatments was tested in mice by monitoring the struggle response latency increase in a nociceptive mechanical stimulus applied to the paw. The subcutaneous (sc) administration of loperamide (4 mg/kg) induced a minimal (nonsignificant) increase in the struggle response latency in comparison to the values from mice treated with its solvent (Figure 3A). The administration of S1RA alone did not change the response to the mechanical stimulus, in agreement with previous studies,^51−53^ but significantly increased the antinociceptive effect induced by loperamide and did so only in the paw injected with the σ_1_R antagonist (Figure 3A). The administration of compounds **5** and **11** did not have any effect *per se* but increased the antinociceptive effect of loperamide at the injected paw, mirroring the effects induced by S1RA (Figure 3A). Therefore, the association of loperamide with any of these three σ_1_R ligands resulted in a synergistic (supra-additive) antinociceptive effect. As the behavioral response was only altered when mice were stimulated in the paw injected with the drug, these antinociceptive effects cannot be attributed to unspecific sedative effects. The coadministration of the σ_1_R agonist PRE-084 (75 μg) with S1RA to loperamide-treated mice completely abolished the effect of the σ_1_R antagonist, and the response latency remained at the level of animals treated with the opioid agonist alone (Figure 3B). The systemic administration of naloxone (1 mg/kg, sc) resulted in a full reversion of the antinociceptive effect of the combination of loperamide + S1RA (Figure 3B). Altogether, these results show that both σ_1_R antagonism and opioid agonism are acting in conjunction for the effect induced by the association of S1RA and loperamide and are in full agreement with previous studies using diverse combinations of σ_1_R antagonists and opioid agonists, which include loperamide but also centrally penetrant opioid analgesics.^54,51−53^ Importantly, these effects of PRE-084 and naloxone on the potentiation of loperamide-induced antinociception by the prototypic σ_1_R antagonist S1RA were identical when using compounds **5** or **11** instead of S1RA (Figure 3B). These data strongly support that compounds **5** and **11** are σ_1_ receptor antagonists.

### 2.2 Molecular Modeling

#### 2.2.1 Docking Studies

**Figure 4:**
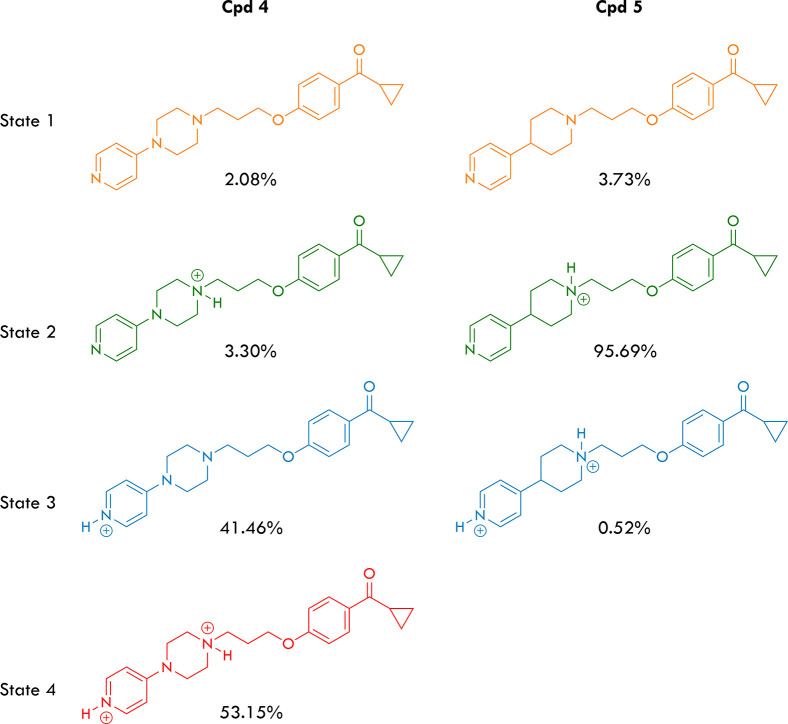
Protonation states and calculated percentages for compounds **4** and **5** at pH 7.4.

All derivatives containing the 4-(pyridin-4-yl)piperazin-1-yl core showed significantly lower affinity toward σ_1_R than those containing the piperidine core (Table 1). When compounds **4** and **5**, which differ only in the piperazine/piperidine core are compared, it becomes evident that their different inhibitory potency must be ascribed to a change in the protonation state or states at physiological pH. Therefore, we calculated the protonation states of compounds **4** and **5** at pH 7.4 using the Marvin software to evaluate this behavior. The results, summarized in Figure 4, suggest that compound **4** exists in nearly equal amounts of the monoprotonated (state 3) and diprotonated (state 4) forms in an aqueous solution. Conversely, compound **5** is found almost exclusively in the monoprotonated form (state 2). The protonation at the pyridine nitrogen in compound **4** can be easily rationalized due to the electron-releasing effect of the amino group in the *para* position. This effect has two consequences: it increases the availability of the electron donor present on the pyridine nitrogen atom (increasing its basicity; DMAP p*K*_a_ = 9.2 vs pyridine p*K*_a_ = 5.2) and, at the same time, reduces the availability of the electron donor present on the nitrogen atom 1 of the piperazine system (decreasing its basicity; 1,4-dimethylpiperazine p*K*_a_ = 8.4). Considering that the literature describes ligands that can be found in the active site of a protein target in doubly protonated state,^55,56^ we have conducted the docking of the compounds containing the piperazine moiety, considering them in state 4 of Figure 4.

The docking studies to identify and evaluate the critical molecular interactions involved in σ_1_R/ligand recognition previously conducted by our group used the Autodock 4.2 scoring function.^57−62^ Unfortunately, this scoring function does not seem suitable for the derivatives with the 4-(pyridin-4-yl) piperazin-1-yl core due to the overestimating docked poses value. To overcome this scoring problem, we used Smina software,^63^ a fork of AutoDock Vina, customized to better support the development of the scoring function to obtain high performance on calculating the free energy of binding. The form and parametrization of scoring functions vary widely between implementations. Force field-based scoring functions seek to quantify the actual molecular forces between a protein and a small molecule. van der Waals, electrostatic, and hydrogen bond interactions are standard components of force field-based scoring functions.^63^ The default Smina scoring function was trained to optimize pose prediction, affinity prediction, and speed simultaneously.^64^ It consists of three steric terms, a hydrogen bond term, a torsion count factor, and a hydrophobic term. However, a larger space of energetic terms was considered in the design of Smina, and these terms remain accessible within the source code. To improve the standard scoring function of Smina (which is the same as that for Autodock Vina) for this set of molecules, we have implemented it by rewarding the poses that formed the saline bridge with Glu172. Furthermore, given the different protonation states, we have modified the desolvation term, which is evaluated using the general approach of Wesson and Eisenberg, in which each type of atom gives a different contribution to this energy, depending on how polar or hydrophobic it is. The approach used here was chosen to satisfy the experimental *K*_i_ values with the calculated ones. For this purpose, we calibrated the Smina scoring function employing compounds **1**–**14**, and **16**–**20** (Table 1); moreover, we test the effective efficiency of this new calibrated function by docking well-known σ_1_ and σ_2_ receptor inhibitors suitably chosen to cover a range of 4 orders of magnitude (Table S1). The 2D plot of the linear regression analysis between experimental and calculated σ_1_ and σ_2_ binding constants values obtained using the calibrated scoring function is provided in Figure S1 and shows a coefficient of determination of 0.983 and 0.998 for the training and the test set, respectively. The new scoring function also reflected the experimental *K*_i_ values obtained for H_3_ receptor. The van der Waals, H-acceptor, H-donor, and solvation terms with their respective calibrated coefficients for the Smina scoring function are reported in Table 2.

**Table 2:** Parameters Used for the Smina Scoring Function

parameter	value
gauss (*o* = 0, *w* = 0, *c* = 8)	–0.035579
gauss (*o* = 3, *w* = 2, *c* = 8)	–0.005156
repulsion (*o* = 0, *c* = 8)	0.840245
hydrophobic (*g* = 0.5, *b* = 1.5, *c* = 8)	–0.035069
non dir h bond (*g* = −0.7, *b* = 0, *c* = 8)	–0.587439
num tors div	1.923
vdw (*i* = 4, *j* = s = 0, ^ = 100, *c* = 8)	0.0003
acceptor acceptor quadratic (*o* = 0, *c* = 8)	–1.5
donor donor quadratic (*o* = 0, *c* = 8)	–2.0
ad4_solvation	0.01148

The calculated free energies of binding (Δ*G*) and *K*_i_ values at the binding site of the σ_1_, σ_2_, and H_3_ receptors for compounds **1**–**20** are reported in Table 3. *In silico* determination of free energies of binding and constants of binding generally agree with experimental data. However, despite the excellent correlation between the *in silico* and experimental data, when evaluating new compounds, one must remember that such results might be a consequence of overfitting. Nevertheless, the compound ordering based on *K*_i_ is preserved. Predictions of ligand binding properties were worse for H_3_R, where some compounds were wrongly evaluated as possessing higher *K*_i_ values than were experimentally determined, such as **11** (156.41 vs. 6.20 nM), **12** (389.31 vs. 22.0 nM), and **16** (297.14 vs. 12.7 nM).

**Table 3:** Calculated Free Energies of Binding, Δ*G* (kcal/mol), and Constants of Binding, *K*_i_ (nM), for the Binding Sites of σ_1_, σ_2_, and H_3_ Receptors for Compounds **1**–**20**

compd	calcd Δ*G* σ_1_	calcd *K*_i_ σ_1_	exptl *K*_i_ σ_1_	calcd Δ*G* σ_2_	calcd *K*_i_ σ_2_	exptl *K*_i_ σ_2_	calcd Δ*G* H_3_	calcd *K*_i_ H_3_	exptl *K*_i_ H_3_
**1**	–8.4	691.27	591.56 ± 94.05	–10.0	46.37	64.27 ± 14.01	–9.75	70.73	40.4 [17.1, 95.9]
**2**	–9.5	107.88	112.20 ± 9.45	–9.3	151.22	129.72 ± 8.23	–10.06	41.90	16.0 [8.1, 31.7]
**3**	–7.8	1904.01	1409.29 ± 312.05	–8.8	351.80	246.60 ± 39.40	–10.22	31.98	10.2 [3.6, 29.0]
**4**	–7.9	1608.17	1531.09 ± 275.32	–9.6	91.12	100.69 ± 15.38	–10.62	16.28	3.17 [2.56, 3.91]
**5**	–11.6	3.11	3.64 ± 0.74	–10.6	16.84	22.44 ± 4.13	–11.22	5.91	7.70 [3.62, 16.38]
**6**	–8.5	583.86	638.26 ± 120.16	–9.4	127.72	108.14 ± 19.16	–10.95	9.32	21.1 [3.8, 116]
**7**	–7.5	3159.97	2958.01 ± 893.58	–9.8	65.0	75.16 ± 13.04	–11.16	6.54	7.86 [2.82, 21.90]
**8**	–8.4	691.27	726.11 ± 176.82	–10.1	39.17	29.24 ± 1.81	–10.9	39.83	3.12 [0.66, 14.60]
**9**	–8.5	583.86	641.21 ± 87.87	–10.0	46.37	32.36 ± 3.46	–11.97	1.67	23.0 [12.4, 42.4]
**10**	–8.0	1358.30	1309.18 ± 331.5	–9.3	151.22	164.06 ± 34.58	–11.66	2.81	5.84 [3.35, 10.19]
**11**	–11.7	2.63	4.41 ± 0.58	–9.7	76.96	67.92 ± 7.51	–9.28	156.41	6.20 [1.90, 20.40]
**12**	–10.8	12.01	14.76 ± 1.86	–9.5	107.88	96.16 ± 14.71	–8.74	389.31	22.0 [6.0, 83.0]
**13**	–10.0	46.37	51.8 ± 9.1	–9.0	250.97	175.4 ± 35.1	–10.21	32.53	37.8 [24.0, 59.4]
**14**	–8.9	297.14	285.1 ± 50.4	–9.4	127.72	100.90 ± 19.3	–9.33	143.75	120 [63, 230]
**15**	–6.5	17102.46	>10000	–7.8	1904.01	1794.73 ± 414.98	–9.7	76.96	115 [26.8, 493]
**16**	–10.1	39.17	37.8 ± 16.1	–9.1	211.97	150.7 ± 26.4	–8.9	297.14	12.7 [4.4, 36.9]
**17**	–8.8	351.80	247.74 ± 30.90	–9.5	107.88	110.15 ± 15.87	–10.09	39.83	16.9 [8.0, 36.0]
**18**	–8.9	297.14	255.27 ± 48.03	–8.9	297.14	178.65 ± 27.16			
**19**	–8.5	583.86	408.3 ± 111.0	–10.0	46.37	59.7 ± 11.2			
**20**	–9.2	179.04	274.16 ± 40.39	–9.9	54.90	65.92 ± 10.98			
S1RA	–10.39	24.00	17.0	–6.83	9795.90	9300			>10000
PRE084	–11.50	3.68	2.2	–6.59	14691.11	>10000			>10000
RHM-4	–7.75	2071.76	2150	–11.99	1.61	0.26			
PIT	–12.22	1.09	0.5	–11.03	8.14	6.5	–10.71	13.98	1.0–2.4

**Figure 5:**
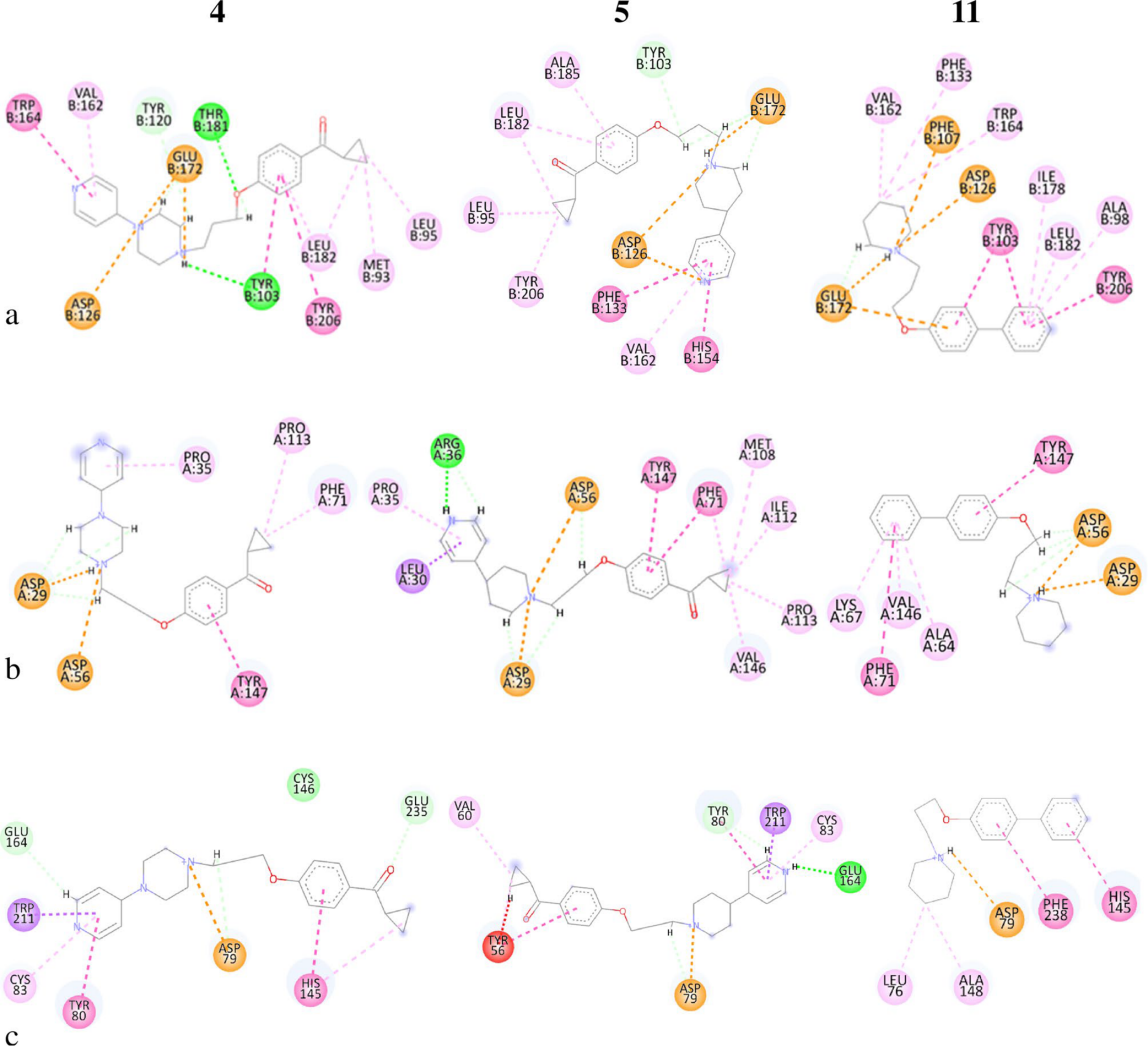
Two-dimensional ligand–receptor interaction diagram of compounds **4**, **5**, and **11** for (a) σ_1_R, (b) σ_2_R, (c) H_3_R.

Compounds **4**, **5**, and **11** have been chosen to describe the interactions with the active sites of the receptors as they present good binding affinity at all three receptors simultaneously (except for the interaction of **4** with σ_1_R). The 2D docked poses of compounds **4**, **5**, and **11** with σ_1_R are reported in Figure 5a (the 3D data are placed in the Supporting Information). The docked poses show that all three compounds form the salt bridge with the Glu172 residue and electrostatic interaction with the Asp126 residue. Furthermore, all three compounds establish numerous interactions with the hydrophobic portion of the receptor active site; particularly, compound **5** has an additional interaction with the Phe133 residue of the π–π type, which probably improves the binding properties with respect to compound **4**.

Regarding the σ_2_R, the poses were discriminated against according to the salt bridge formation with the residue Asp56. The poses 2D with the σ_2_R receptor for compounds **4**, **5**, and **11** are shown in Figure 5b (3D data, see the Supporting Information). In addition to the formation of the salt bridge with Asp56, the three compounds possess a further electrostatic interaction with the residue Asp29; furthermore, compound **5** makes a hydrogen bond of 2.11 Å with Arg36, which is missing in compound **4**. This is due to the presence of the piperazine ring, which involves a different geometric arrangement compared to compound **5**, which instead possesses a piperidine ring (Figure 5). All three compounds engage in numerous hydrophobic interactions, and compound **4** has one less π–π interaction with residue Phe71 than compounds **5** and **11**. The lack of these two interactions probably makes it less active toward the σ_2_R. The key to discriminate the best poses for the H_3_R was the formation of the salt bridge with the Asp79 residue. The 2D poses of ligands **4**, **5**, and **11** with the H_3_R receptor are shown in Figure 5c (the 3D data are placed in the Supporting Information). Compounds **4** and **5** have several interactions in common in addition to that with the Asp79 residue; both interact in the same way with the Trp211 and Cys83 residues and differently with the Tyr80 and Glu164. In particular, compound **5** establishes a conventional hydrogen bond of 2.08 Å with Glu164 and a hydrogen bond between the carbon of pyridine and Tyr80 (2.41 Å). Compound **4** establishes a π–π interaction with Tyr80 and an unconventional C–H hydrogen bond (the pyridine C_2_) with the Glu164 residue (2.25 Å). Furthermore, the carbonyl group establishes another unconventional C–H bond with the Glu235 residue (2.14 Å). Compound **11**, with respect to **4** and **5**, has fewer electrostatic interactions but more hydrophobic interactions with the Leu76, Ala148, Phe238, and His145 residues.

#### 2.2.2 Molecular Dynamics Simulations

**Figure 6:**
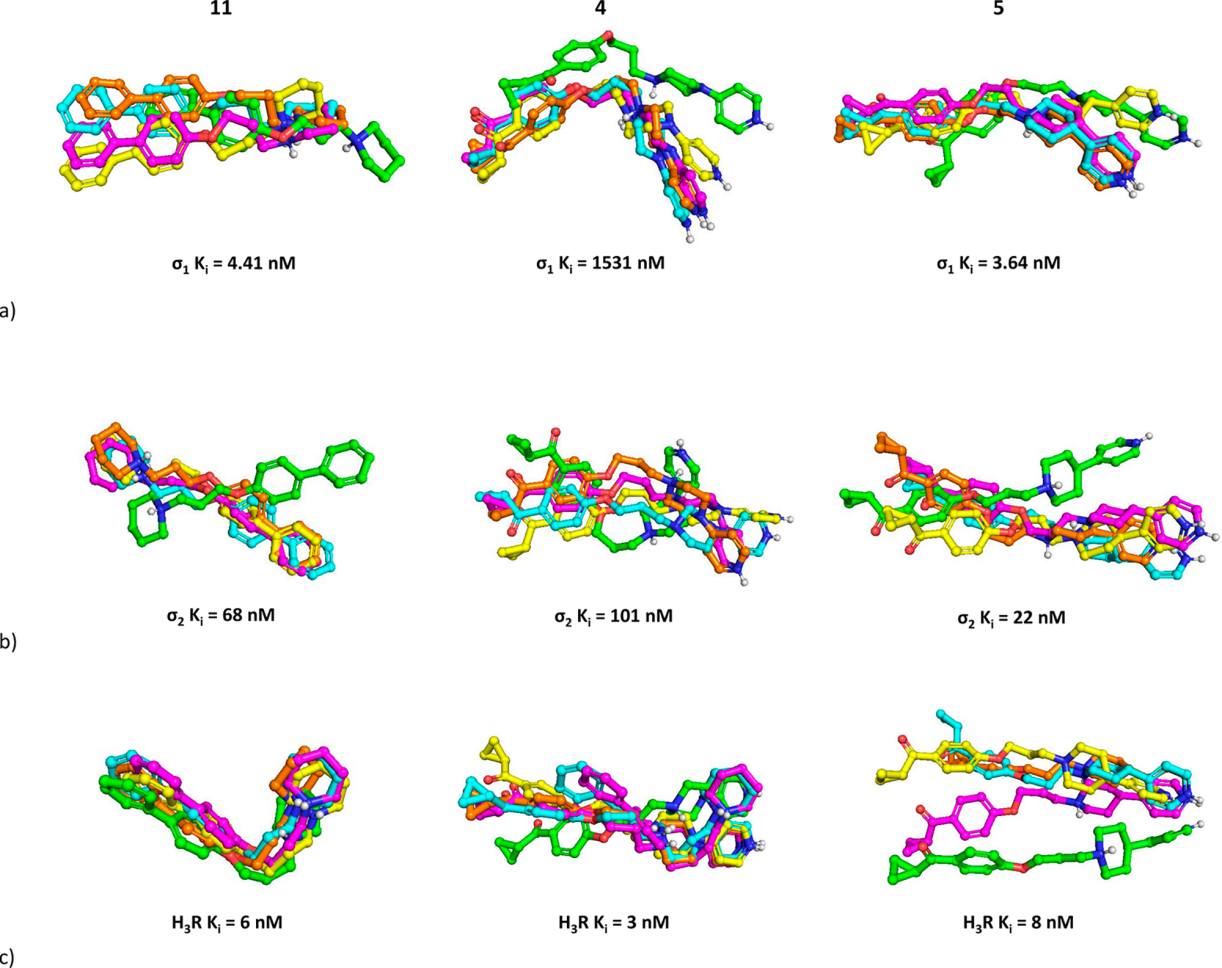
Examination of compound stability in the binding pocket for (a) σ_1_R, (b) σ_2_R, and (c) H_3_R. Compound poses were captured at following frames: 1, green; 250, yellow; 500, magenta; 750, cyan; 1000, orange.

**Figure 7:**
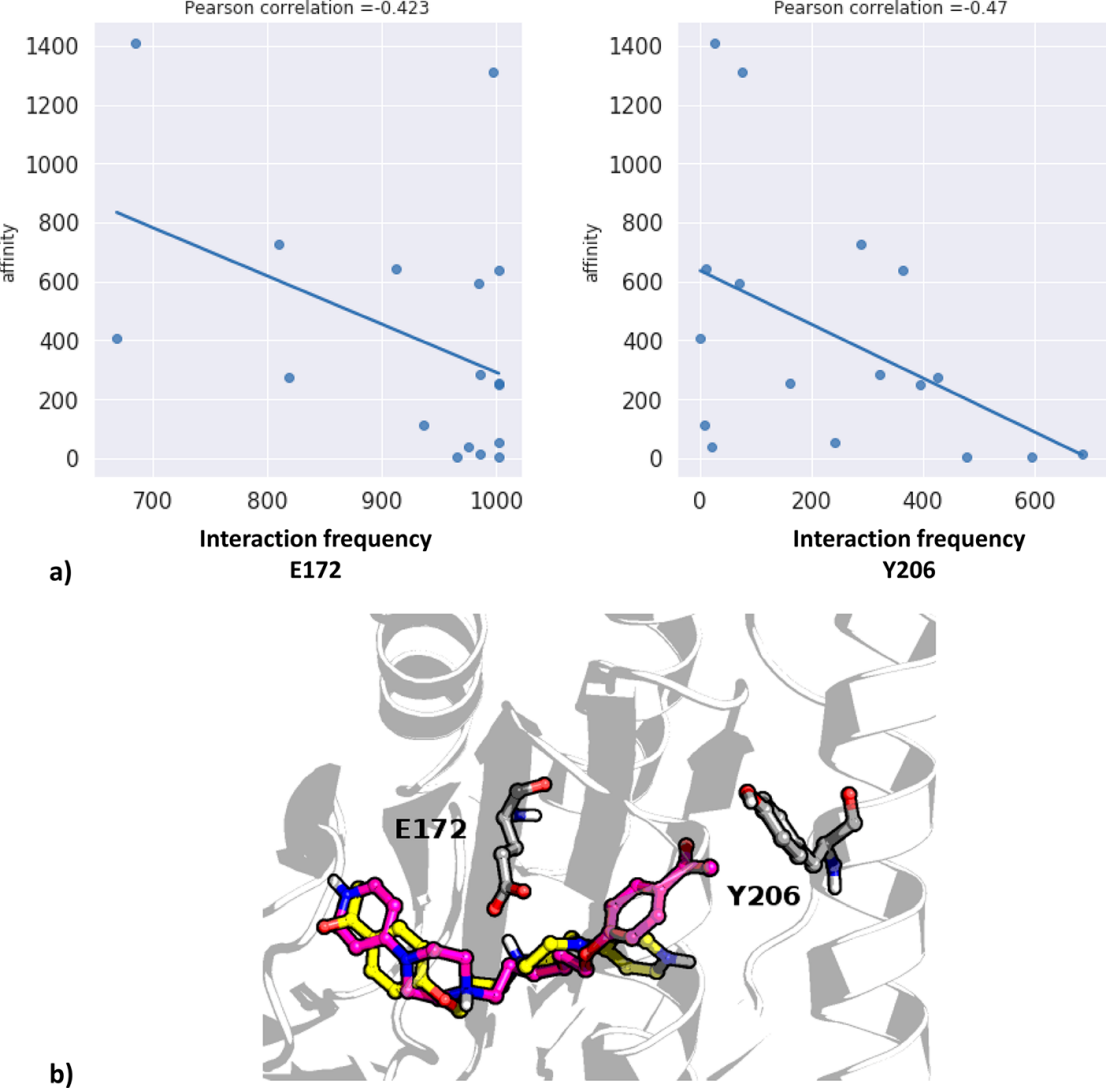
Outcome of correlational studies between frequency of interaction of ligands with particular amino acid residues of σ_1_R: (a) Pearson correlation coefficients for the highest correlated residues, (b) visualization of the highest correlated residues with examples of docked compounds: **3**, yellow; **16**, magenta.

To examine the compound pose stability and correlate experimental outcome with the interaction frequency of particular amino acids, molecular dynamics (MD) simulations were carried out for each compound with all three receptors (software Desmond, duration 500 ns). The docking poses were used as starting points for simulations. The results were examined in two ways: first, the stability of compounds in the binding pocket during MD was analyzed in relation to their affinities (Figure 6). Then, the interaction frequency between modeled compounds and each amino acid of the target protein was correlated with compound affinity to detect positions that might help to explain the observed structure–activity–interaction relationships. The correlations were expressed via Pearson’s correlation coefficient, and the results for σ_1_R are presented in Figure 7. The remaining data are presented in the Supporting Information.

Examination of Figure 6 indicates that we can search for a correlation between compound pose stability during MD simulation and compound affinity only for the σ_1_R. The rate of conformational changes of the most active compounds **5** and **11** (*K*_i_ values toward σ_1_R below 5 nM) was much lower than that of compound **4** with *K*_i_ over 1500 nM, although **5** and **11** also changed their initial orientations. On the other hand, atom positions of all analyzed ligands varied when σ_2_R was considered. Compounds **5** and **11** with the lowest affinities changed their initial positions and remained rather stably oriented during the rest of the simulation. Interestingly, **4**, with only a slightly higher *K*_i_ value toward σ_1_R, fluctuated during the whole simulation, although the compound occupied the same area of the binding pocket for the whole time. When MD simulations toward H_3_R are analyzed, **11** was very stably fitted in the H_3_R binding site, with almost imperceptible changes in atom positions. In contrast, the poses of **4** and **5**, which were also very active toward this receptor, varied significantly for the subsequent simulation frames. Examination of the frequency of particular ligand–protein interactions with reference to the compound activity revealed that there are some amino acid residues for which a tendency of decreasing or increasing contact frequency can be correlated with the compound affinity. Figure 7 presents the two highest correlated positions: E172 and Y206. Although the Pearson correlation coefficient values are not very high (−0.423 and −0.47, respectively; Figure 7a), there is a noticeable trend that higher affinity (expressed via lower *K*_i_ values) is connected with the increase in the interaction frequency with E172 and Y206. The examination of the position of these amino acids in the protein (Figure 7b) revealed that E172 interacts with the central part of the ligand, whereas the Y206 establishes some contacts with the terminal part. As a result, interaction with tyrosine is significantly less frequent than contacts formed by the glutamic acid, as the orientation and distance of a ligand and Y206 do not always meet the criteria of making a contact. Therefore, the ligand–protein interaction matrix obtained for Y206 is much sparser than the respective contact patterns obtained for the E172 (data are presented in the Supporting Information).

The contact patterns indicated in the correlational studies can be used to design new derivatives of the examined compounds by focusing on the interactions provided with the indicated residues.

## 3 Conclusions

In an attempt to explain recent studies showing that some clinically evaluated H_3_R antagonists possess nanomolar affinity at sigma-1 receptors, we selected 20 representative structures among our previously described H_3_R ligands to investigate their affinity at the σ_1_R and σ_2_R. Interestingly, only six compounds showed higher affinity toward σ_1_R than σ_2_R with the highest binding preference to σ_1_R for compounds **11**, **12**, and **5** (selectivity factor 15.4, 6.5, and 6.2, respectively). Likewise, all these ligands share a common structural feature: the piperidine moiety in their basic part. It is most likely a key structural element for the dual activity toward H_3_ and σ_1_ receptors, as shown by comparing compounds **4** and **5**. The evaluation of more ligands based on the piperidine core will be the subject of our upcoming research as detailed SAR studies are needed in this area. Considering that structures **4** and **5** differ only in the piperazine/piperidine nucleus, we can hypothesize that their different inhibitory potency could be attributed to either a change in the protonation states of the ligand within the receptor site or thermodynamic factors related to the solvation energy of ligands. Recent studies have shown that protonation changes occur upon binding; the complementary change in the degree of buffer ionization can produce significant enthalpy data.^65^ Indeed, unknown protonation events can contribute to the variance of the enthalpy. Given the current limitations of docking software, we decided to modify some parameters in the Smina scoring function to obtain free energies of binding consistent with the experimental data. The advantage of the scoring terms used in this case is based on a complete thermodynamic model, extensible for the use in protein–ligand docking in those cases where the protonation states of the ligand can influence the solvation terms. The performance of the new scoring functions is similar to the existing AutoDock4 force field, which has been proven in our previous studies. Certainly, the piperidine ring has been found as the dominant structural element responsible for the compounds’ activity at the σ_1_R while maintaining the affinity toward the H_3_R and a moderate selectivity profile in terms of the σ_2_R. On the other hand, it has recently been reported that σ_2_R agonists exert a profound effect on mechanical hypersensitivity in the spared nerve injury (SNI) model with a duration of action and potency that is superior to that of gabapentin.^66^ Small molecule modulation of σ_2_R may thus represent a new approach for managing pain by a previously unexplored mechanism of action. However, improving selectivity will be the subject of our further studies, as the main goal of this work is to determine the synergistic effect of dual H_3_R and σ_1_R modulation in the treatment of pain. In the course of the study, we selected compounds **5** and **11** as lead structures and determined their affinity at other histamine receptor subtypes, as well as their agonistic or antagonistic properties at H_3_R and σ_1_R, as this parameter may be crucial for further animal studies. Interestingly, both ligands turned out to be potent histamine H_3_ and σ_1_ receptor antagonists, as evidenced by the results of the mini-G protein recruitment assay, as well as *in vivo* studies. The administration of compounds **5** and **11** enhanced the antinociceptive effect of loperamide, and the simultaneous administration of the σ_1_R agonist PRE-084 was able to significantly reverse the effect of these compounds on loperamide-induced antinociception. Interestingly, a similar tendency to increase the analgesic effect was previously described in the group of aryloxypropanolamines, where replacement of the primary amino group with cyclic structures such as piperidine, pyrrolidine, and morpholine increased the analgesic activity of these compounds.^67^ However, this effect was not observed in the case of piperazine derivatives. Given the high preclinical efficacy of individual selective σ_1_R or H_3_R ligands in various pain models, our research could be of crucial importance in the search for novel, dual-targeting compounds that may contribute to the development of new strategies for the treatment of neuropathic pain. The determination of whether such ligands are more effective when compared to separate selective drugs will be the subject of our future studies.

## 4 Experimental Protocols

Compounds **1**–**20** were obtained within previous studies and used in the biological assays with high purity estimated using LC-MS. Details of their synthesis and analyses are described elsewhere;^45−48^ purity set is provided in the Supporting Information.

### 4.1 Pharmacology

#### 4.1.1 Affinity at σ_1_ and σ_2_ Receptors

Brain and liver homogenates for σ_1_R and σ_2_R binding assays were prepared from male Dunkin–Hartley guinea pigs and Sprague–Dawley rats, respectively (ENVIGO RMS S.R.L., Udine, Italy) as previously reported.^61^*In vitro* σ_1_R ligand binding assays were carried out in Tris buffer (50 mM, pH 7.4) for 150 min at 37 °C. The thawed membrane preparation of guinea pig brain cortex was incubated with increasing concentrations of test compounds and [^3^H](+)-pentazocine (2 nM) in a final volume of 0.5 mL. Unlabeled (+)-pentazocine (10 μM) was used to measure nonspecific binding. Bound and free radioligand were separated by fast filtration under reduced pressure using a Millipore filter apparatus through Whatman GF 6 glass fiber filters, which were presoaked in a 0.5% poly(ethylenimine) water solution. Each filter paper was rinsed three times with ice-cold Tris buffer (50 mM, pH 7.4), dried at rt, and incubated overnight with scintillation fluid in pony vials. The bound radioactivity was determined using a liquid scintillation counter (Beckman LS 6500).^61,68^*In vitro* σ_2_R ligand binding assays were carried out in Tris buffer (50 mM, pH 8.0) for 120 min at rt. The thawed membrane preparation of rat liver was incubated with increasing concentrations of test compounds and [^3^H]DTG (2 nM) in the presence of (+)-pentazocine (5 μM) as σ_1_ masking agent in a final volume of 0.5 mL. Nonspecific binding was evaluated with unlabeled DTG (10 μM). Bound and free radioligand were separated by fast filtration under reduced pressure using a Millipore filter apparatus through Whatman GF 6 glass fiber filters, which were presoaked in a 0.5% poly(ethylenimine) water solution. Each filter paper was rinsed three times with ice-cold Tris buffer (10 mM, pH 8), dried at rt, and incubated overnight with scintillation fluid in pony vials. The bound radioactivity was determined using a liquid scintillation counter (Beckman LS 6500).^69^ The *K*_i_ values were calculated with the program GraphPad Prism 7.0 (GraphPad Software, San Diego, CA, USA). The *K*_i_ values are given as mean value ± CI from at least two independent experiments performed in duplicate.

#### 4.1.2 Affinity at Histamine Receptors

Compounds (as oxalate salts) were tested in H_3_R *in vitro* binding studies, using methods described previously.^47,48^ Ligands were tested at 5 to 11 appropriate concentrations in a [^3^H]*N*^α^-methylhistamine (*K*_D_ = 3.08 nM) radioligand depletion assay to determine the affinity at human recombinant histamine H_3_R stably expressed in HEK293 cells. Radioligand binding experiments at the H_1_R, H_2_R, and H_4_R were performed as previously described in Rosier et al.^70^ and Bartole et al.^71^ with HEK293T-SP-FLAG-hH_*x*_R (*x* = 1, 2, or 4) expressing the respective hHR. The following radioligands and concentrations were used: [^3^H]mepyramine (hH_1_R, *K*_d_ = 5.1 nM, *c* = 5 nM; Novandi Chemistry AB, Södertälje, Sweden), [^3^H]UR-DE257 (hH_2_R, *K*_d_ = 66.9 nM, *c* = 50 nM),^72^ [^3^H]UR-PI294 (hH_4_R, *K*_d_ = 3.6 nM, *c* = 4 nM)^73^. Data represent mean values ± CI from three independent experiments, each performed in triplicate. The normalized competition binding curves were then fitted with a four-parameter logistic fit yielding IC_50_ values using Prism 8.4.3 software (GraphPad, SanDiego, CA). The *K*_i_ values were estimated from the Cheng–Prusoff equation.^74^

#### 4.1.3 Intrinsic Activity toward H_3_R: Mini-G Protein Recruitment Assay

The mini-G protein recruitment assay was performed as previously described.^48^ The assay relies on the split-luciferase complementation technique^49^ and meets the demands of a sufficiently high dynamic range without radioactivity. The mini-G protein assay was performed with living HEK293T cells stably coexpressing human H_3_R with the C-terminal fragment fused to the small fragment of the NanoLuc (SmBit) and the mini-G_i_ protein N-terminally tagged with the large fragment of the NanoLuc (LgBit). Upon activation of the H_3_R, the mini-G_i_ protein was recruited to the receptor, allowing the NanoLuc fragments to form a functional luciferase, giving concentration-dependent luminescence traces in the presence of a substrate. The intensity of the luminescence provided information on the potency and efficacy of test compounds. For agonist activity detection, histamine was used as positive control.

Compound dilution series in three replicates were incubated with a Nano-Glo Live Cell Substrate (furimazine), and emitted light was recorded for 45 min using the EnSpire Multimode Plate Reader (Tecan Austria GmbH). In the case of the antagonist assay, histamine at a single concentration (1 μM) was added to the preincubated mixture of cells in the presence of antagonist in different concentrations and the substrate, and afterward the emitted light was recorded for 45 min.

#### 4.1.4 *In Vivo* Pharmacological Activity

Female CD1 mice (Charles River, Barcelona, Spain) were used in all experiments. The experiments were performed during the light phase (from 9:00 h to 15:00 h). Animal care was provided in accordance with institutional (Research Ethics Committee of the University of Granada, Granada, Spain), regional (Junta de Andalucía, Spain), and international standards (European Communities Council directive 2010/63).

We aimed to test whether compounds **5** and **11** behaved *in vivo* as σ_1_ antagonists or agonists. As reference σ_1_ compounds, we used S1RA (4-[2-[[5-methyl-1-(2-naphthalenyl)-1*H*-pyraol-3-yl]oxy]ethyl]morpholine hydrochloride), a known selective σ_1_ receptor antagonist (DC Chemicals, Shanghai, China), and PRE-084 (2-[4-morpholinethyl]1-phenylcyclohexanecarboxylate hydrochloride; Tocris Cookson Ltd., Bristol, United Kingdom), a selective σ_1_ receptor agonist.^34^ S1RA, PRE-084, and compound **5** were dissolved in sterile physiologic saline (0.9% NaCl). Compound **11** was dissolved in 1% Tween 80 (Sigma-Aldrich, Madrid, Spain) in ultrapure water and heated until dissolved before injection. We previously tested that this solvent did not alter the animals’ behavioral response to the mechanical stimulation (data not shown). All these compounds (or their solvents) were administered intraplantarally (ipl) into the right hind paw in a volume of 20 μL using a 1710 TLL Hamilton microsyringe (Teknokroma, Barcelona, Spain) with a 30^1/2^-gauge needle. The ipl injection was made 5 min before nociceptive testing to minimize systemic absorption of the compounds. When PRE-084 was associated with S1RA, **5**, or **11**, drugs were dissolved in the same solution and injected together to avoid paw lesions from multiple injections.

As it is known that σ_1_ antagonism can enhance opioid antinociception and that σ_1_ agonism reverses this effect,^52^ we tested whether our compounds modulated the antinociceptive effect induced by the opioid agonist loperamide hydrochloride (Sigma-Aldrich). This drug was dissolved in 1% dimethyl sulfoxide (DMSO; Merck KGaA, Darmstadt, Germany) in ultrapure water and injected subcutaneously (sc) into the interscapular area in a volume of 5 mL/kg, 30 min before behavioral testing. Naloxone hydrochloride (Tocris Cookson Ltd.) was used as a standard opioid antagonist^52^ and was dissolved in physiological saline and sc administered 5 min before loperamide injection.

Nociceptive stimulation of the hind paw of the animals was made with an Analgesimeter (model 37215, Ugo-Basile, Varese, Italy) as previously described.^52^ After drug administration, mice were gently pincer grasped between the thumb and index fingers by the skin above the interscapular area. Then, a blunt cone-shaped paw-presser was applied at a constant intensity of 450 g to the dorsal surface of the hind paw until the animal showed a struggle response. The struggle latency was measured with a chronometer. Evaluations were done twice alternately to each hind paw at intervals of 1 min between stimulations.

Statistical analysis was carried out with the two-way analysis of variance (ANOVA), followed by a Bonferroni post hoc test. ANOVA was performed with the SigmaPlot 12.0 program. The differences between values were considered significant when the *P*-value was below 0.05.

### 4.2 Molecular Modeling

#### 4.2.1 Structure Preparation and Minimization

The structures of all the molecules used in this study were built using Marvin Sketch (18.24, ChemAxon Ltd., Budapest, Hungary). A first molecular mechanics energy minimization was used for 3D structures created from the SMILES; the Merck molecular force field (MMFF94) present in Marvin Sketch^75^ was used. The protonation states were calculated, assuming a neutral pH. The PM3 Hamiltonian, as implemented in the MOPAC package (MOPAC2016 v. 18.151, Stewart Computational Chemistry, Colorado Springs, CO, USA),^76,77^ was then used to further optimize the 3D structures^75^ before the alignment for the docking calculations.

#### 4.2.2 Docking Studies

Flexible ligand docking experiments were performed employing AutoDock Smina software^63^ with their respective coefficients to the Smina scoring function (Table 3), using the crystal structure of the human σ_1_ receptor model bound to PD144418 (PDB 5HK1) retrieved from the PDB_REDO Data Bank. Docking for the σ_2_ receptor and the H_3_R receptor was performed using the homology models previously built by the same authors.^78,45^

#### 4.2.3 Molecular Dynamics Simulations

All ligand–receptor complexes obtained in the docking procedure constituted input for MD simulations. The simulations were carried out in Desmond using the TIP3P solvent model and POPC as a membrane model (the receptor was automatically placed in the membrane during the setup preparation). The OPLS3e force field was used, and a pressure of 1.01325 bar was applied. Simulations were run at 300 K. The box shape was orthorhombic with a size of 10 Å × 10 Å × 10 Å. Each simulation lasted 500 ns, and ligand–protein contacts occurring during MD were analyzed using the Simulation Interaction Diagram facility present in the Schrödinger Suite.

## Supporting Information Available

Purity confirmation data, chemistry methods, calibration of parameters for the Smina scoring function, molecular modeling of 3D docking poses of compounds **4**, **5**, and **11** in the binding site of σ_1_R, σ_2_R, and H_3_R, outcome of correlational studies between frequency of interaction of ligands with particular amino acid residues of σ_2_R, ^1^H and ^13^C NMR spectra of compounds **11** and **12**, and HPLC traces of all the final compounds (PDF)
