# Supplementary material for: Structural and Molecular Insight into Piperazine and Piperidine Derivatives as Histamine H3 and Sigma-1 Receptor Antagonists with Promising Antinociceptive Properties
Source: ACS Chem Neurosci. 2021 Dec 15;13(1):1–15. doi: 10.1021/acschemneuro.1c00435 (PMC8739840; doi:10.1021/acschemneuro.1c00435)

Supplementary Material

Structural and molecular insight into piperazine and piperidine derivatives as histamine H_3 and sigma-1 receptor antagonists with promising antinociceptive properties

Katarzyna Szczepańska[+ a,b], Sabina Podlewska[+ a,b,*], Maria Dichiara [c], Davide Gentile [c], Vincenzo Patamia [c], Niklas Rosier [d], Denise Mönnich [d], M[a] Carmen Ruiz Cantero [e], Tadeusz Karcz [a], Dorota Łażewska [a], Steffen Pockes [d], Agata Siwek [f], Enrique J. Cobos [e], Agostino Marrazzo [c], Holger Stark [g], Antonio Rescifina [c], Andrzej J. Bojarski [b], Emanuele Amata [c,*], Katarzyna Kieć-Kononowicz [a,*]

[a] *Department of Technology and Biotechnology of Drugs, Faculty of Pharmacy, Jagiellonian University Medical College, Medyczna 9, Kraków 30-688, Poland*
[b] *Maj Institute of Pharmacology, Polish Academy of Sciences, Smętna 12, Kraków 31-343, Poland*
[c] *Department of Drug and Health Sciences, University of Catania, V.le A. Doria, 95125 Catania, Italy*
[d] *Institute of Pharmacy, Faculty of Chemistry and Pharmacy, University of Regensburg, Universitätsstraße 31, D-93053 Regensburg, Germany*
[e] *Department of Pharmacology and Neurosciences Institute (Biomedical Research Center), University of Granada, and Biosanitary Research Institute ibs.GRANADA, Avenida de la Investigación 11, 18016 Granada, Spain*
[f] *Department of Pharmacobiology, Faculty of Pharmacy, Jagiellonian University Medical College, Medyczna 9, Kraków 30-688, Poland*
[g] *Institute of Pharmaceutical and Medicinal Chemistry, Heinrich Heine University Düsseldorf, Universitaetsstr. 1, 40225 Duesseldorf, Germany*

* Corresponding authors. E-mail addresses: smusz@if-pan.krakow.pl (Sabina Podlewska), mfkonono@cyf-kr.edu.pl (Katarzyna Kieć-Kononowicz), eamata@unict.it (Emanuele Amata).
[+] These authors contributed equally to this work.

Contents

1. Purity confirmation data S2
2. Chemistry S3
3. Calibration of parameters for the Smina scoring function S4
4. Molecular modeling – 3D docking poses of compounds 4, 5, and 11 in the binding site of σ_1R, σ_2R, and H_3R S5
5. Outcome of correlational studies between frequency of interaction of ligands with particular amino acid residues of σRs S6
6. References S8
7. ^{1}H- and ^{13}C-NMR spectra of compounds 11 and 12. HPLC traces of all the final compounds S9

1. Purity confirmation data

Mass spectra (LC/MS) were performed on Waters TQ Detector mass spectrometer. High-resolution mass spectra (HRMS) were performed on UltrafleXtreme maldi-tof/tof mass spectrometer. HPLC analyses were performed on Waters Alliance 2695 Separations Module with Waters 2998 Photodiode Array Detector (Chromolith® SpeedROD RP-18 endcapped 50-4.6 HPLC column was used)[1–4]. Elemental analyses (C, H, N) were performed on an Elemental AnalyserVarioEl III (Hanau, Germany) and agreed with theoretical values within ± 0.4%[1,2].

Cpd	LC/MS			HRMS	RP-HPLC			References
	Purity [%]	Retention time [min]	ESI m/z $[M+H]^+$	MALDI m/z $[M+H]^+$	Purity [%]	Retention time [min]	k	
**1**	100	3.23	326	326.217	99.21	0.923	1.64	3
**2**	100	4.02	355	-	99.30	1.082	2.09	1
**3**	100	1.87	341	-	100	0.723	1.07	2
**4**	100	3.04	366	366.203	100	0.756	1.16	4
**5**	100	3.28	365	365.209	100	0.788	1.25	4
**6**	100	3.89	374	374.224	99.64	1.111	2.17	3
**7**	100	3.67	399	399.234	99.40	0.971	1.77	4
**8**	97.83	3.18	402	402.212	95.42	0.971	1.77	3
**9**	97.28	4.07	436	436.183	95.56	1.040	1.97	4
**10**	95.61	3.61	420	420.195	95.76	0.928	1.65	4
**11**	100	5.24	296	296.209	100	1.280	2.66	-
**12**	100	4.47	324	324.201	100	1.140	2.26	-
**13**	100	4.18	369	-	100	1.168	2.34	1
**14**	98.46	4.66	383	-	98.06	1.252	2.58	1
**15**	100	2.26	355	-	-	-	-	2
**16**	93.73	2.90	382	382.253	92.19	0.942	1.69	3
**17**	100	3.43	396	396.266	100	1.029	1.94	3
**18**	100	5.26	396	396.303	100	1.334	2.81	3
**19**	94.79	3.69	410	410.284	94.47	1.124	2.21	3
**20**	100	4.03	424	424.296	100	1.202	2.43	3

Cpd 2: Anal. Calcd for $C_{22}H_{31}N_3O$ x $C_2H_2O_4$: C, 64.99, N, 9.47, H, 7.50%. Found: C, 64.96, N, 9.52, H, 7.48.

Cpd 13: Anal. Calcd for $C_{23}H_{33}N_3O$ x $C_2H_2O_4$: C, 65.62, N, 9.18, H, 7.71%. Found: C, 65.60, N, 9.19, H, 7.72.

Cpd 14: Anal. Calcd for $C_{24}H_{35}N_3O$ x $C_2H_2O_4$: C, 66.22, N, 8.91, H, 7.91%. Found: C, 66.19, N, 8.89, H, 7.91.

Cpd 15: Anal. Calcd for $C_{21}H_{27}N_3O_2$ x $C_2H_2O_4$: C, 62.29, N, 9.47, H, 6.59%. Found: C, 62.32, N, 9.49, H, 6.63.

2. Chemistry

Compounds **11** and **12** were obtained by *N*-alkylation of piperidine with proper bromides by the method described by Kuder *et al.*[5] Both compounds have been previously described in the literature[6,7].

*(Biphenyl-4-yloxy)propyl)piperidine hydrogen oxalate (**11**)*
4-(3-Bromopropoxy)-1,1'-biphenyl (CAS113795-28-1) (1.46 g; 5 mmol), piperidine (0.21 g; 2.5 mmol) was refluxed for 24 h in the mixture of ethanol-water (5:1) with the powdered potassium carbonate (0.52 g; 3.8 mmol) and catalytic amount of potassium iodide. After purification oily product was converted into hydrogen oxalate. White solid, yield (28%), m.p. 213-215 °C, $C_{22}H_{27}NO_5$ (MW=385.46). ^{1}H NMR (500 MHz, DMSO-d_6) δ ppm: 7.54 - 7.59 (m, 4H), 7.36 - 7.42 (m, 2H), 7.27 (tt, *J*=7.30, 1.15 Hz, 1H), 6.96 - 7.01 (m, 2H), 4.05 (t, *J*=6.01 Hz, 2H), 3.02 - 3.16 (m, 4H), 2.46 - 2.47 (m, 2H), 2.04 - 2.14 (m, 2H), 1.68 (br. s., 4H), 1.49 (br. s., 2H). ^{13}C NMR (DMSO-d_6): 164.85, 158.43, 140.27, 133.34, 129.42, 128.32, 126.71, 115.48, 65.62, 54.09, 52.82, 24.22, 23.36, 22.10. LC-MS: purity 100% t_R= 5.24, (ESI) *m/z* $[M+H]^+$ 296.20.

*Phenyl (4-(3-(piperidin-1-yl)propoxy)phenyl)phenyl)methanone hydrogen oxalate (**12**)*
4-(3-Bromopropoxy)phenyl)(phenyl)methanone (CAS108357-63-7) (0.998 g; 3.85 mmol), piperidine (0.328g; 3.85 mmol) was refluxed for 24 h in the mixture of ethanol-water (5:1) with the powdered potassium carbonate (0.69 g; 5 mmol) and catalytic amount of potassium iodide. After purification oily product was converted into hydrogen oxalate. White solid, yield (45%), m.p. 129-132 °C, $C_{23}H_{27}NO_6$ (MW=413.47). ^{1}H NMR (300 MHz, DMSO-d_6) δ ppm: 7.69 - 7.73 (m, 2H), 7.60 - 7.66 (m, 3H), 7.49 - 7.54 (m, 2H), 7.03 - 7.08 (m, 2H), 4.11 (t, *J*=6.16 Hz, 2H), 2.95 - 3.19 (m, 6H), 2.08 - 2.17 (m, 2H), 1.65 - 1.74 (m, 4H), 1.49 (br. s., 2H). ^{13}C NMR (DMSO-d_6): 194.97, 165.21, 162.52, 138.25, 132.73, 129.79, 129.01, 114.90, 66.01, 53.85, 52.67, 23.94, 23.18, 22.06. LC-MS: purity 100% t_R= 4.47, (ESI) *m/z* $[M+H]^+$ 324.18.

None of the final structures has the Pan-assay interference compounds properties[8].

3. Calibration of parameters for the Smina scoring function

Table S1. Well-known σ1 and σ2 receptor ligands used to test the new calibrated Smina scoring function, and their experimental and calculated binding constant values.

Compound	Exp. σ_1 K_i (nM)	Calcd K_i σ_1 (nM)	Exp. σ_2 K_i (nM)	Calcd K_i σ_2 (nM)
Ifenprodil	1.02	2.93	18.8	26.62
PRE-084	2.2	3.68	13091	14691.11
Haloperidol	2.6	3.24	77	89.57
Pentazocine	4.3	6.30	1465	1285.65
BD1063	13.7	16.70	204	231.24
S1RA	17	24.00	9300	9795.90
DTG	124	102.65	18	22.23
F-ISO	330	357.87	6.95	8.35
RHM-4	2150	2071.76	0.26	1.61
Pitolisant	0.5	1.09	6.5	8.14

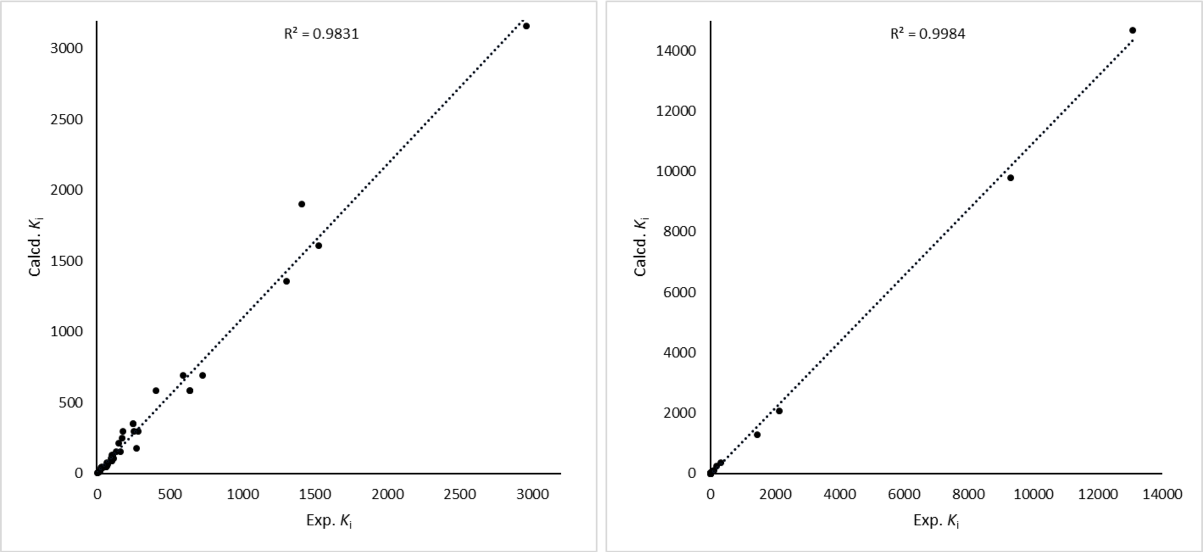

Figure S1. 2D plot of the linear regression fit between experimental and calculated σ1 and σ2 binding constants values for (left) compounds **1**–**14**, and **16**–**20** (the training set), reported in **Table 1**, and (right) those of the chosen well-known ligands (the test set), reported in Table **S1**. All values have been calculated by the calibrated Smina scoring function employing the optimized parameters reported in **Table 2**.

4. Molecular modeling – 3D docking poses of compounds 4, 5, and 11 in the binding site of σ_1R, σ_2R, and H_3R

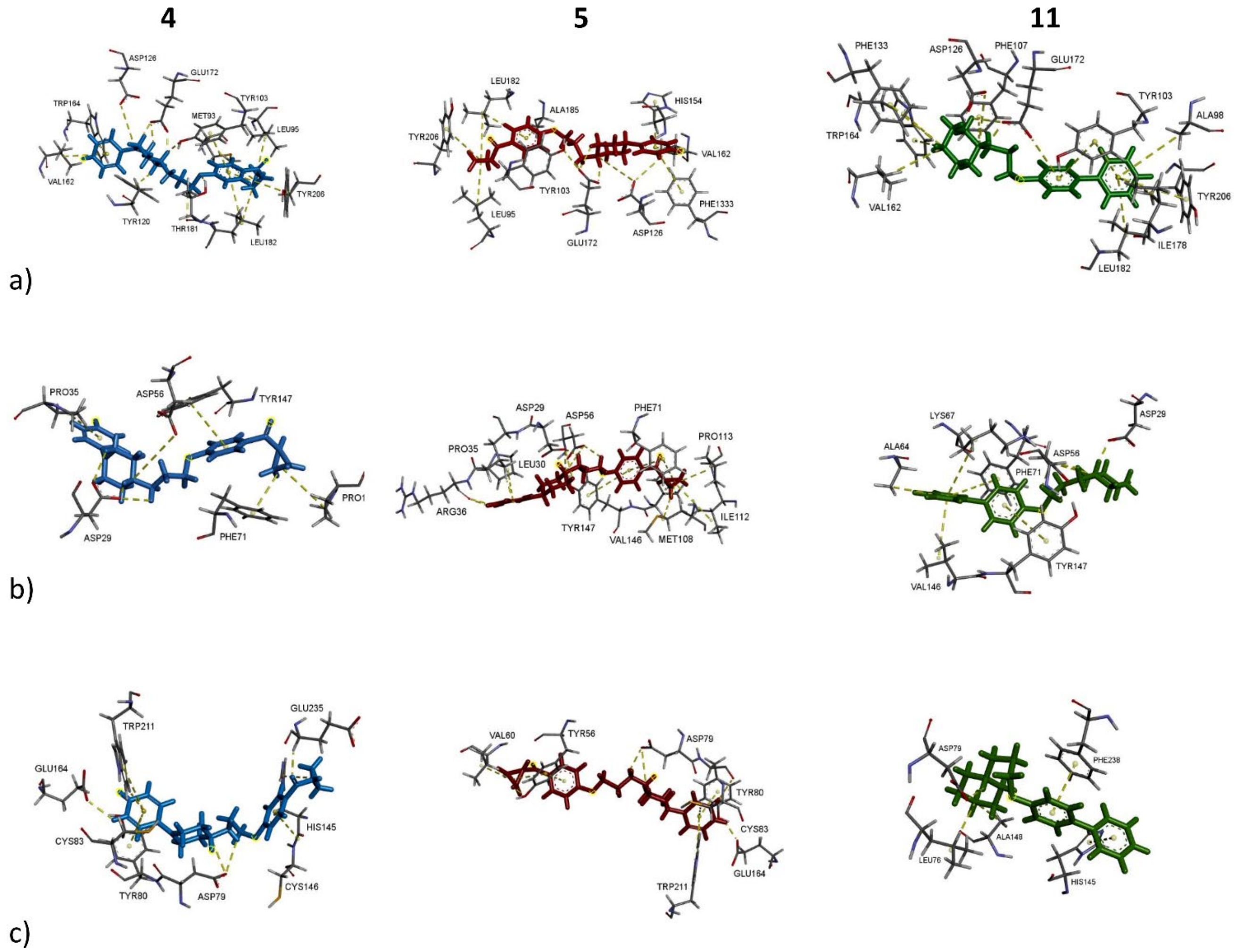

Figure S2. 3D docking poses of compounds **4**, **5**, and **11** in the binding site of a) σ_1R, b) σ_2R, c) H_3R.

5. Outcome of correlational studies between frequency of interaction of ligands with particular amino acid residues of σRs

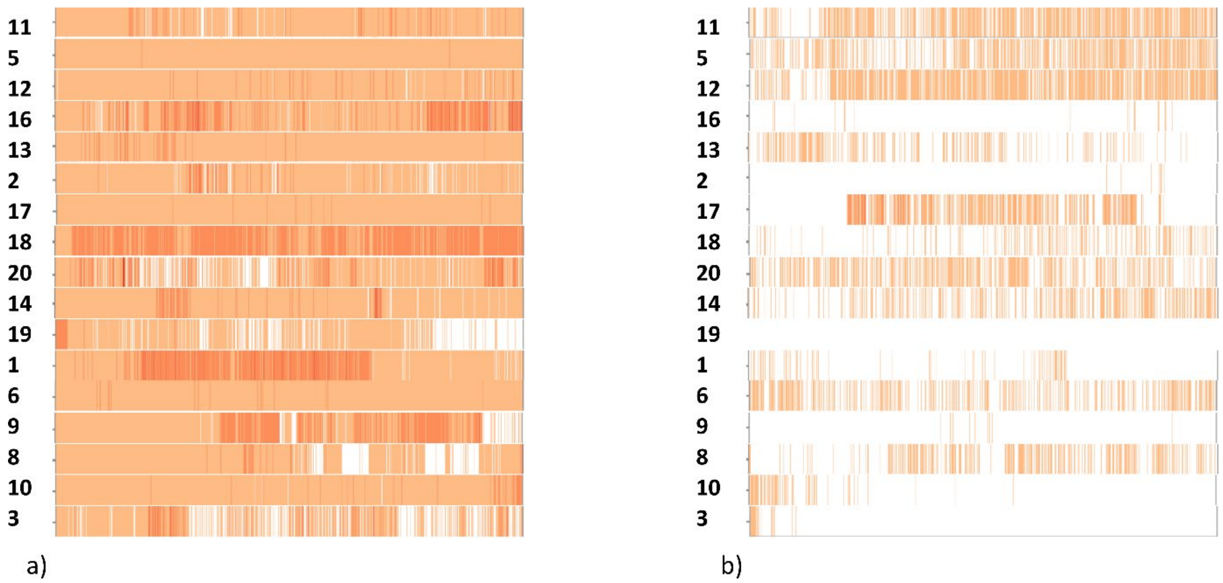

Figure S3. Ligand-protein interaction diagrams obtained during MD simulations for the highest correlated residues of σ_1R: a) E172, b) Y206.

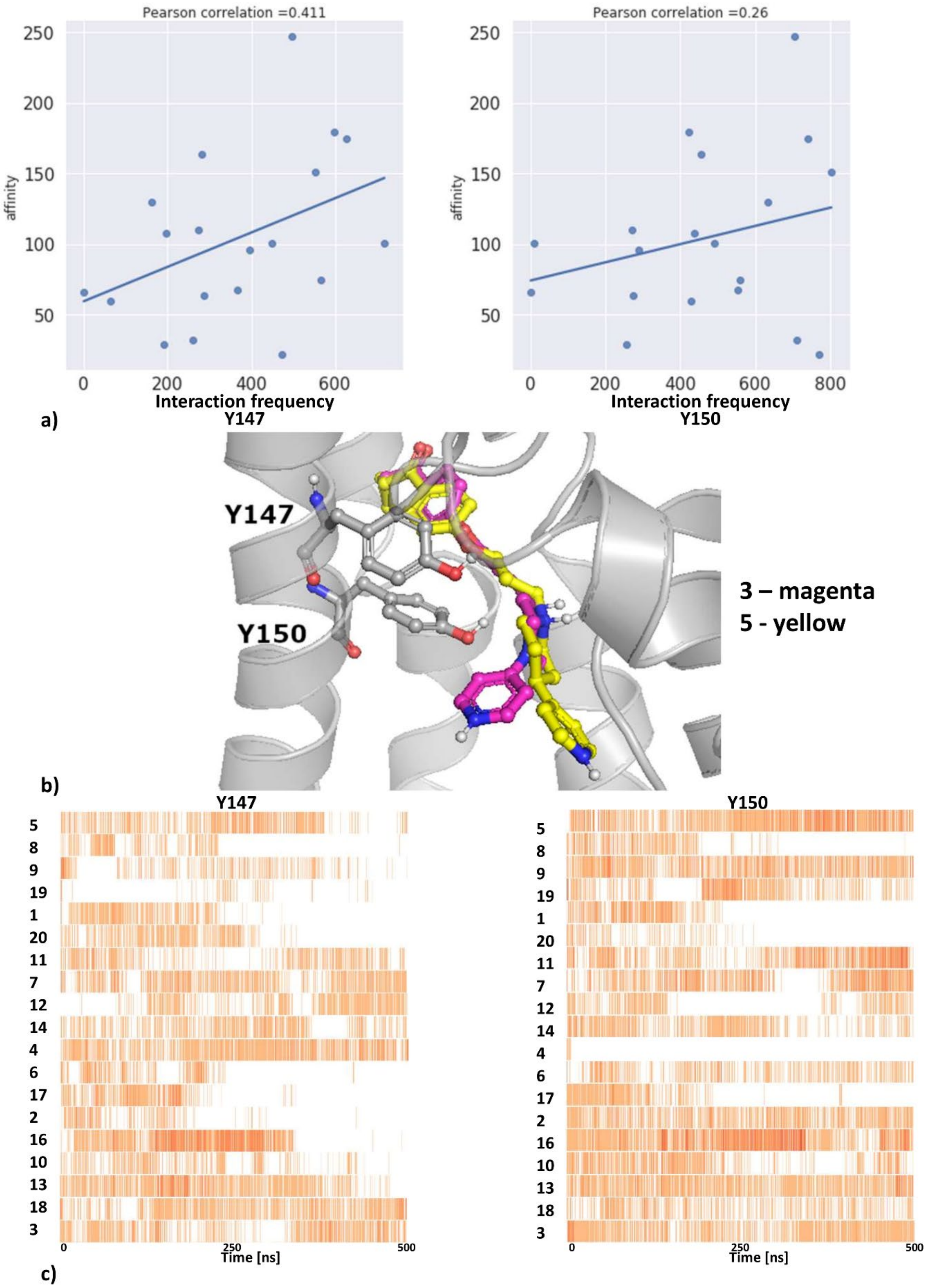

Figure S4. Outcome of correlational studies between frequency of interaction of ligands with particular amino acid residues of σ_2R, a) Pearson correlation coefficients for the highest correlated residues, b) visualization of the highest correlated residues with examples of docked compounds, c) ligand-protein interaction diagrams obtained during md simulations for the highest correlated residues.

6. References

1. Szczepańska K, Karcz T, Mogilski S, Siwek A, Kuder KJ, Latacz G, Kubacka M, Hagenow S, Lubelska A, Olejarz A, Kotańska M, Sadek B, Stark H, Kieć-Kononowicz K. Synthesis and biological activity of novel *tert*-butyl and *tert*-pentylphenoxyalkyl piperazine derivatives as histamine H_3R ligands. *Eur J Med Chem*. 2018; 152: 223-234.

2. Szczepańska K, Karcz T, Kotańska M, Siwek A, Kuder KJ, Latacz G, Mogilski S, Hagenow S, Lubelska A, Sobolewski M, Stark H, Kieć-Kononowicz K. Optimization and preclinical evaluation of novel histamine H_3 receptor ligands: Acetyl and propionyl phenoxyalkyl piperazine derivatives. *Bioorganic Med Chem*. 2018; 26(23-24): 6056-6066.

3. Szczepańska K, Karcz T, Siwek A, Kuder KJ, Latacz G, Bednarski M, Szafarz M, Hagenow S, Lubelska A, Olejarz-Maciej A, Sobolewski M, Mika K, Kotańska M, Stark H, Kieć-Kononowicz K. Structural modifications and *in vitro* pharmacological evaluation of 4-pyridyl-piperazine derivatives as an active and selective histamine H_3 receptor ligands. *Bioorg Chem*. 2019; 103071.

4. Szczepańska K, Pockes S, Podlewska S, Höring C, Mika K, Latacz G, Bednarski M, Siwek A, Karcz T, Nagl M, Bresinsky M, Mönnich D, Seibel U, Kuder KJ, Kotańska M, Stark H, Elz S, Kieć-Kononowicz K. Structural modifications in the distal, regulatory region of histamine H_3 receptor antagonists leading to the identification of a potent anti-obesity agent. *Eur J Med Chem*. 2020; 113041. doi:10.1016/j.ejmech.2020.113041.

5. Kuder K, Łażewska D, Latacz G, Schwed JS, Karcz T, Stark H, Karolak-Wojciechowska J, Kieć-Kononowicz K. Chlorophenoxy aminoalkyl derivatives as histamine H_3R ligands and antiseizure agents. *Bioorganic Med Chem*. 2016, 24(2): 53-72.

6. Bertrand I, Capet M, Lecomte JM, Levoin N, Ligneau X, Poupardin-Olivier O, Robert P, Schwartz JC, Labeeuw O. BIOPROJET. Phenoxypropylpiperidines and -pyrrolidines and their use as histamine H_3 -receptor ligands. WO2006117609 (2006)

7. Levoin N, Labeeuw O, Calmels T, Poupardin-Olivier O, Berrebi-Bertrand I, Lecomte JM, Schwartz JC, Capet M. Novel and highly potent histamine H3 receptor ligands. Part 1: Withdrawing of hERG activity. *Bioorganic Med Chem Lett*. 2011, 21(18): 5378-5383.

8. Baell JB, Holloway GA. New substructure filters for removal of pan assay interference compounds (PAINS) from screening libraries and for their exclusion in bioassays. *J Med Chem*. 2010, 53(7): 2719-2740.

7. ^{1}H- and ^{13}C-NMR spectra of compounds 11 and 12. HPLC traces of all the final compounds

This report was created by ACD/NMR Processor Academic Edition. For more information go to www.acdlabs.com/nmrproc/

Cpd11

Acquisition Time (sec)	3.4918	***Date***	30 Oct 2020 10:57:56			***Date Stamp***	30 Oct 2020 10:57:08
File Name	C:\Users\turbo\Desktop\E377_905_33_PROTON-1-1.jdf	***Frequency (MHz)***	500.16	***Nucleus***	1H	***Number of Transients***	8
Origin	ECA	***Original Points Count***	32768	***Owner***	delta	***Points Count***	32768
Pulse Sequence	proton.jxp	***Solvent***	DMSO-d6	***Spectrum Offset (Hz)***	3251.0396	***Sweep Width (Hz)***	9384.38
Temperature (degree C)	22.500						

^{1}H NMR (500 MHz, DMSO-d_6) δ ppm 1.49 (br. s., 2 H) 1.68 (br. s., 4 H) 2.04 - 2.14 (m, 2 H) 2.46 - 2.47 (m, 2 H) 3.02 - 3.16 (m, 4 H) 4.05 (t, *J*=6.01 Hz, 2 H) 6.96 - 7.01 (m, 2 H) 7.27 (tt, *J*=7.30, 1.15 Hz, 1 H) 7.36 - 7.42 (m, 2 H) 7.54 - 7.59 (m, 4 H)

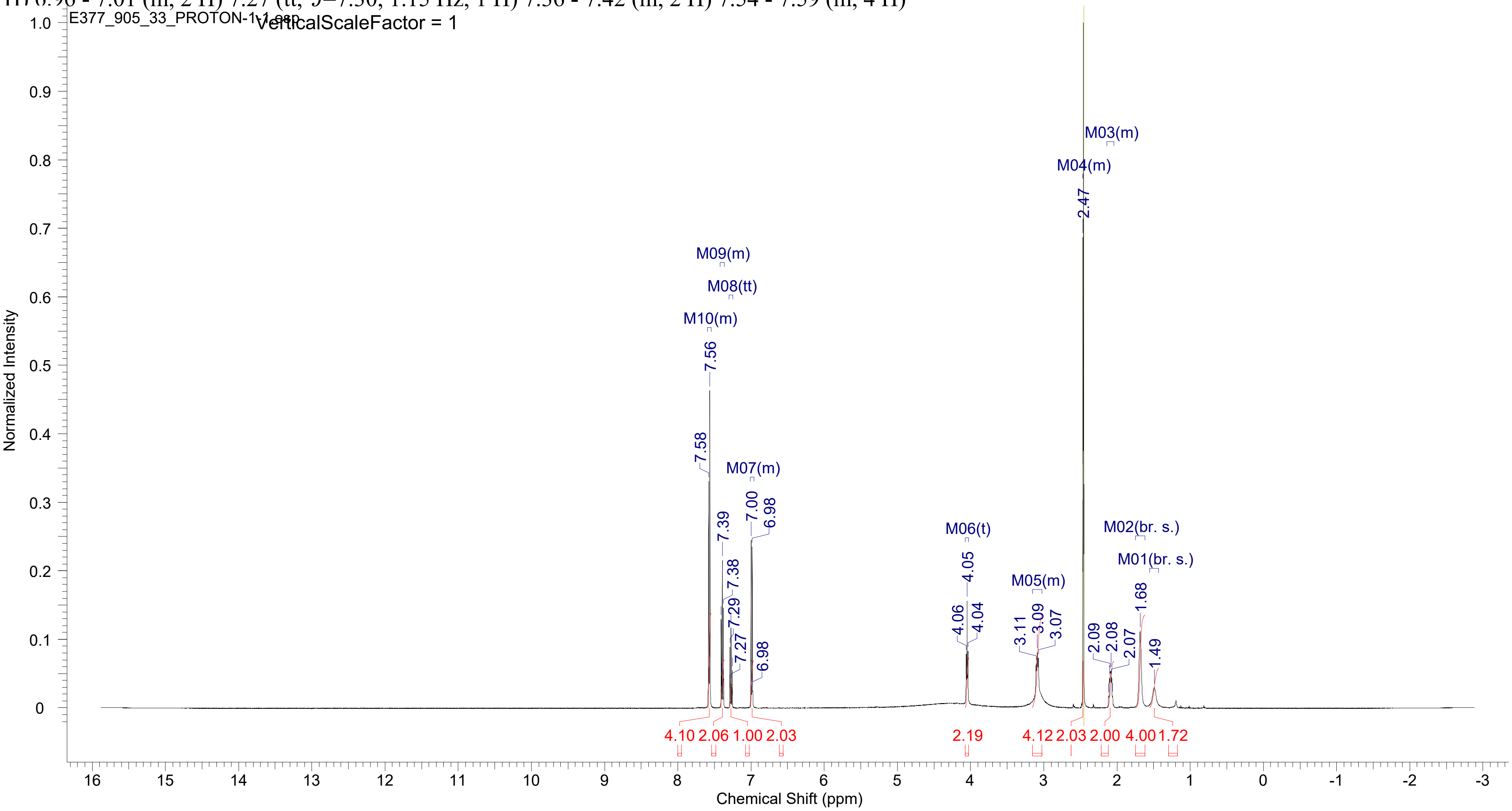

This report was created by ACD/NMR Processor Academic Edition. For more information go to www.acdlabs.com/nmrproc/

Cpd11

Acquisition Time (sec)	1.6568	***Date***	30 Oct 2020 11:50:08			***Date Stamp***	30 Oct 2020 10:58:27
File Name	C:\Users\turbo\Desktop\E377_905_33_CARBON-1-1.jdf			***Frequency (MHz)***	125.77	***Nucleus***	13C	***Number of Transients***	1024
Origin	ECA	***Original Points Count***	65536	***Owner***	delta	***Points Count***	65536	***Pulse Sequence***	carbon.jxp
Solvent	DMSO-d6	***Spectrum Offset (Hz)***	12576.5293	***Sweep Width (Hz)***	39556.96	***Temperature (degree C)***	22.500		

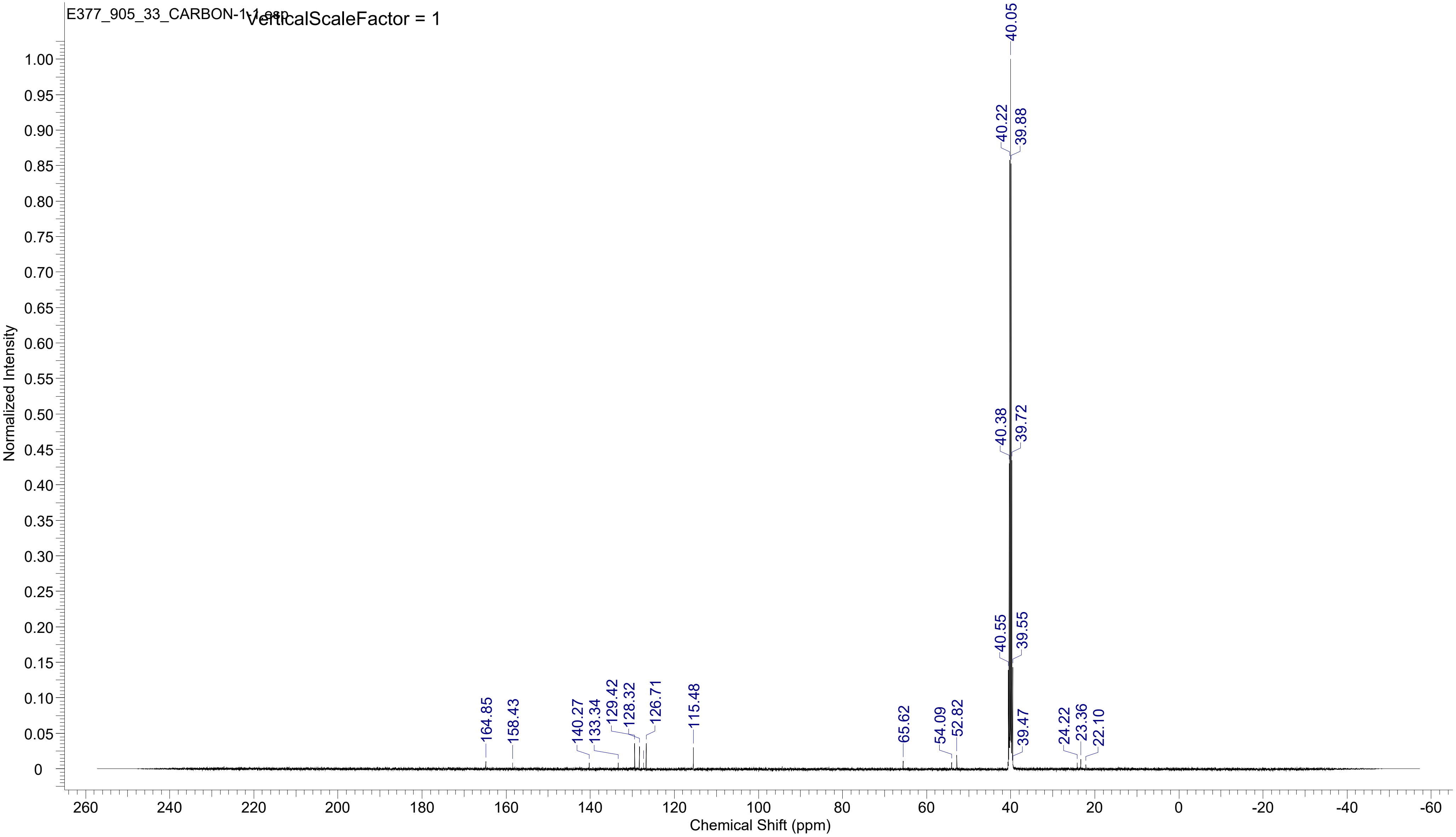

This report was created by ACD/NMR Processor Academic Edition. For more information go to www.acdlabs.com/nmrproc/

Cpd12

Acquisition Time (sec)	3.4918	***Date***	24 Jun 2021 08:39:15			***Date Stamp***	24 Jun 2021 08:38:27
File Name	C:\Users\turbo\Desktop\W232_905_33_PROTON-1-1.jdf					***Frequency (MHz)***	500.16	***Nucleus***	1H
Number of Transients	8	***Origin***	ECA	***Original Points Count***	32768	***Owner***	delta	***Points Count***	32768
Pulse Sequence	proton.jxp	***Solvent***	DMSO-d6	***Spectrum Offset (Hz)***	3251.0396	***Sweep Width (Hz)***	9384.38	***Temperature (degree C)***	21.000

^{1}H NMR (500 MHz, DMSO-d_6) δ ppm 1.49 (br. s., 2 H) 1.65 - 1.74 (m, 4 H) 2.08 - 2.17 (m, 2 H) 2.95 - 3.19 (m, 6 H) 4.11 (t, *J*=6.16 Hz, 2 H) 7.03 - 7.08 (m, 2 H) 7.49 - 7.54 (m, 2 H) 7.60 - 7.66 (m, 3 H) 7.69 - 7.73 (m, 2 H)

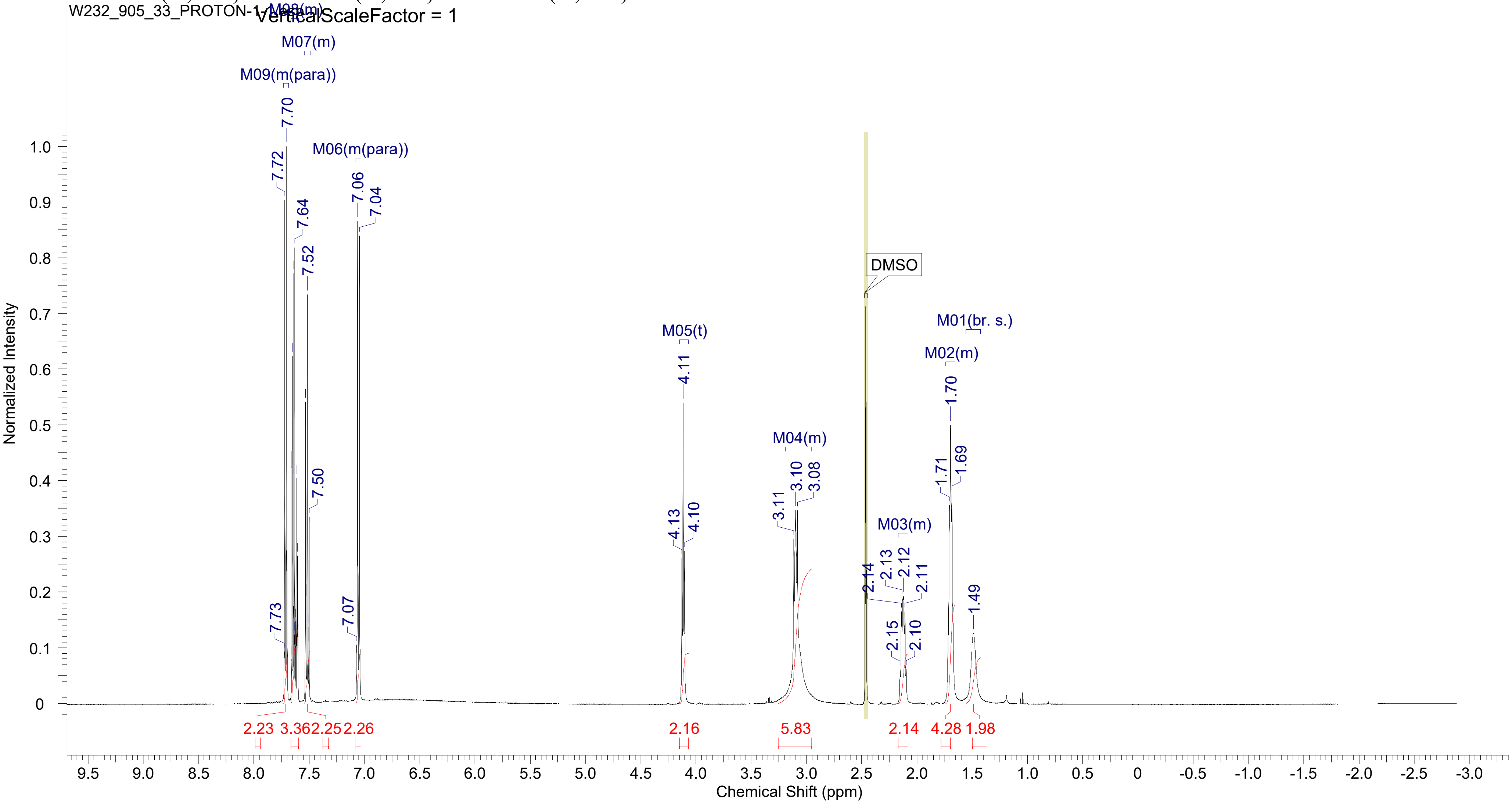

This report was created by ACD/NMR Processor Academic Edition. For more information go to www.acdlabs.com/nmrproc/

Cpd12

Acquisition Time (sec)	1.6568	***Date***	24 Jun 2021 09:31:35			***Date Stamp***	24 Jun 2021 08:39:46
File Name	C:\Users\turbo\Desktop\W232_905_33_CARBON-1-1.jdf	***Frequency (MHz)***	125.77	***Nucleus***	13C	***Number of Transients***	1024
Origin	ECA	***Original Points Count***	65536	***Owner***	delta	***Points Count***	65536
Pulse Sequence	carbon.jxp	***Solvent***	DMSO-d6	***Spectrum Offset (Hz)***	12576.5293	***Sweep Width (Hz)***	39556.96
Temperature (degree C)	21.000						

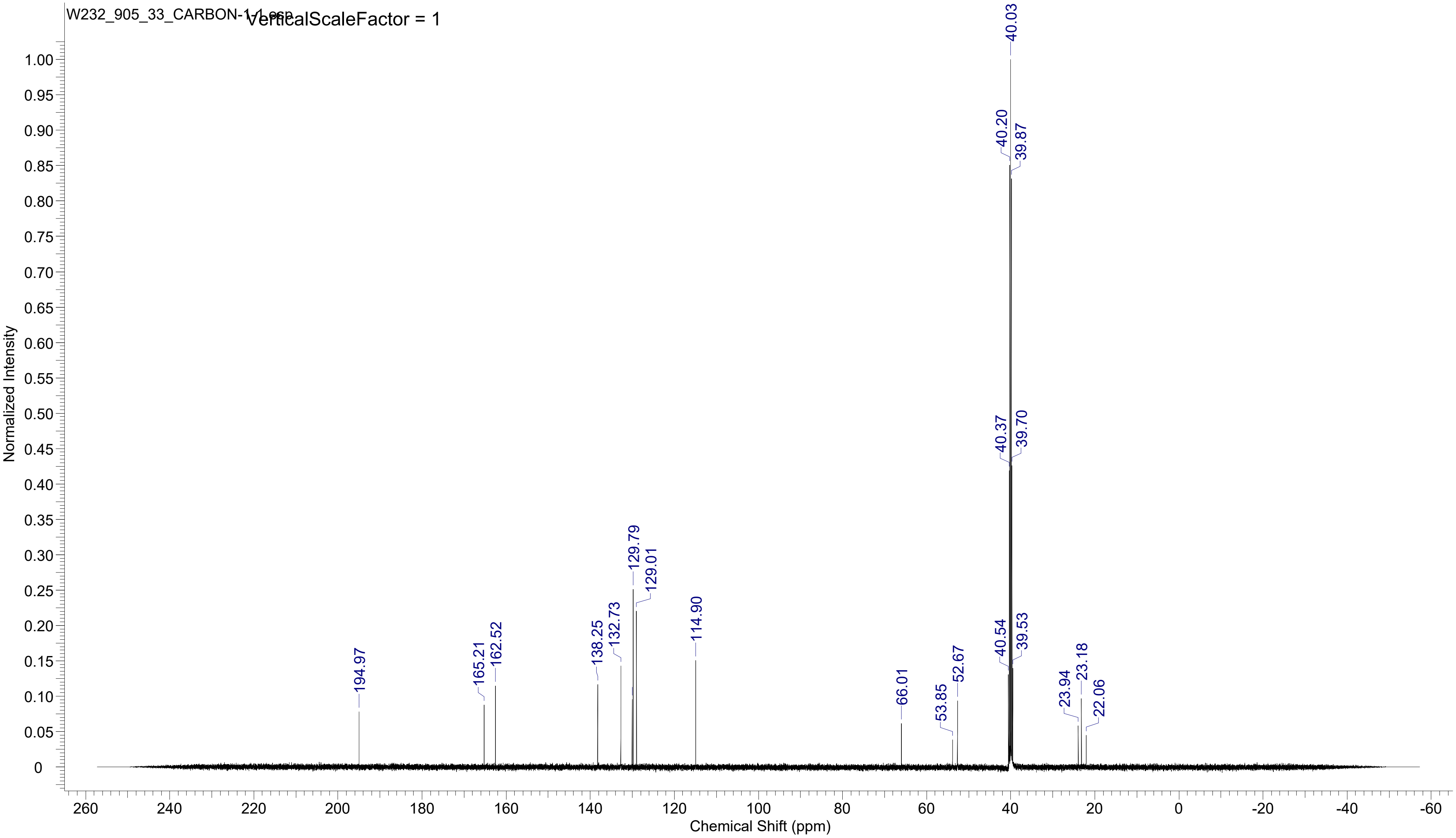

Cpd1

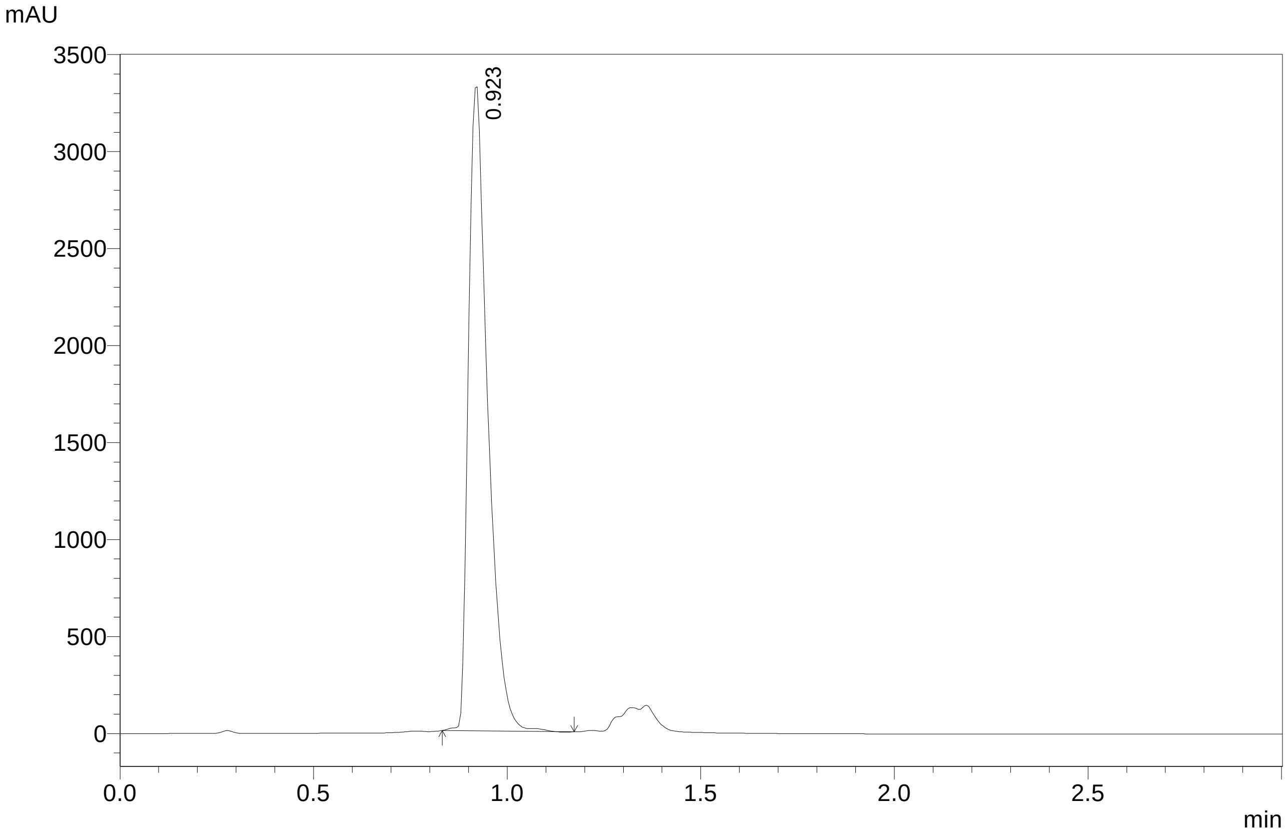

mAU
3500
3000
2500
2000
1500
1000
500
0
0.923
0.0
0.5
1.0
1.5
2.0
2.5
min

SHIMADZU LabSolutions Cpd2

<Chromatogram>

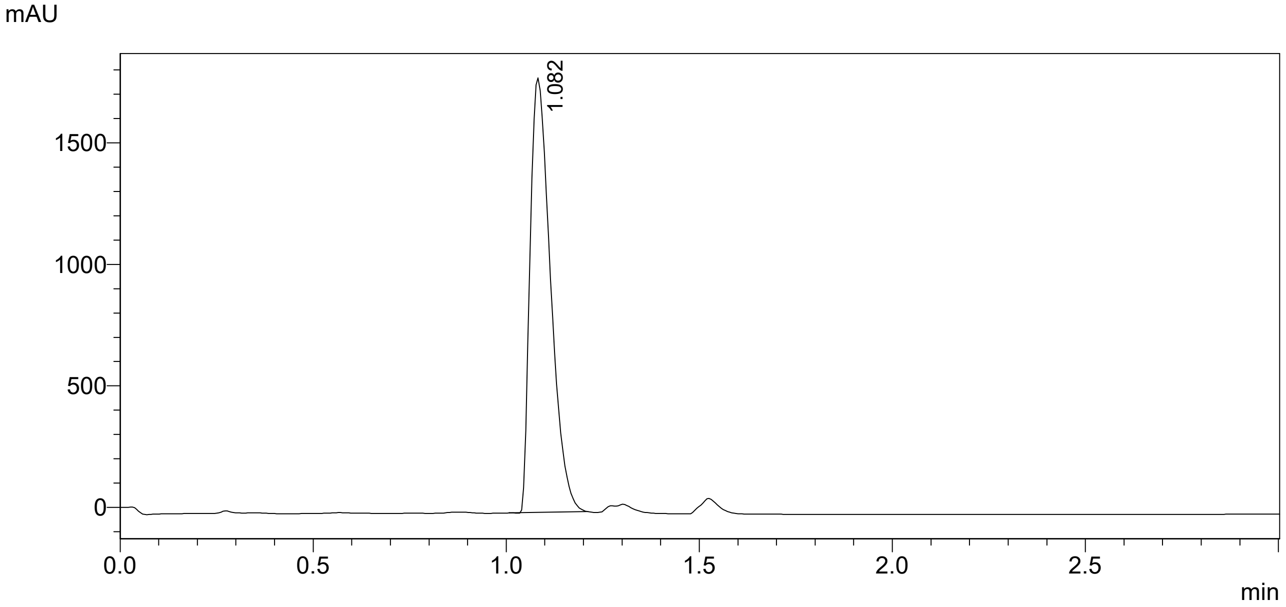

Cpd3

<Chromatogram>

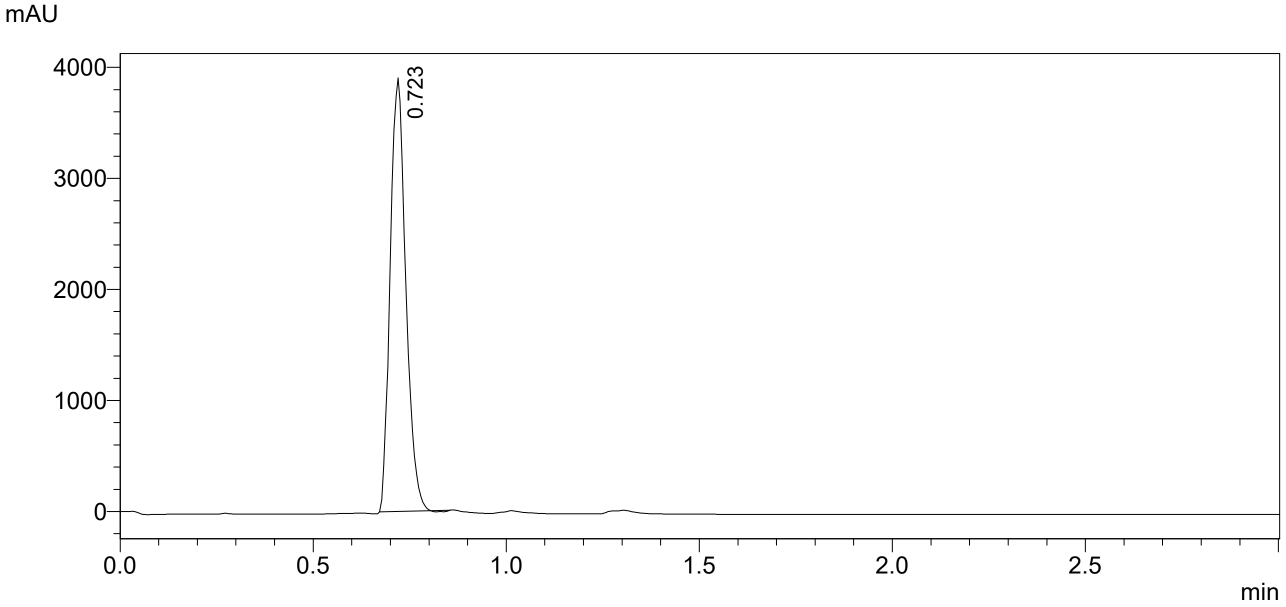

Cpd4

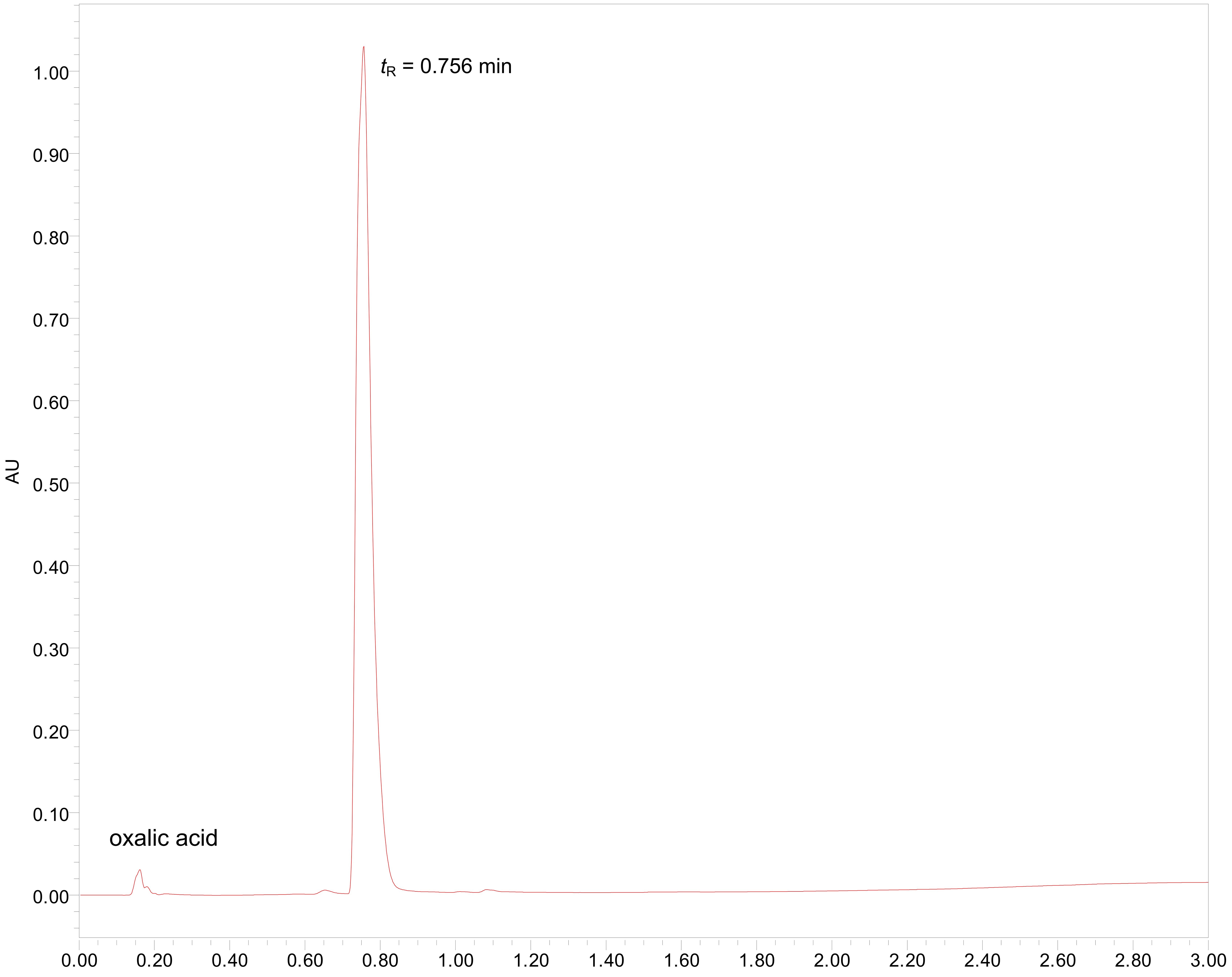

t_R = 0.756 min
oxalic acid
AU
1.00
0.90
0.80
0.70
0.60
0.50
0.40
0.30
0.20
0.10
0.00
0.00 0.20 0.40 0.60 0.80 1.00 1.20 1.40 1.60 1.80 2.00 2.20 2.40 2.60 2.80 3.00

Cpd5

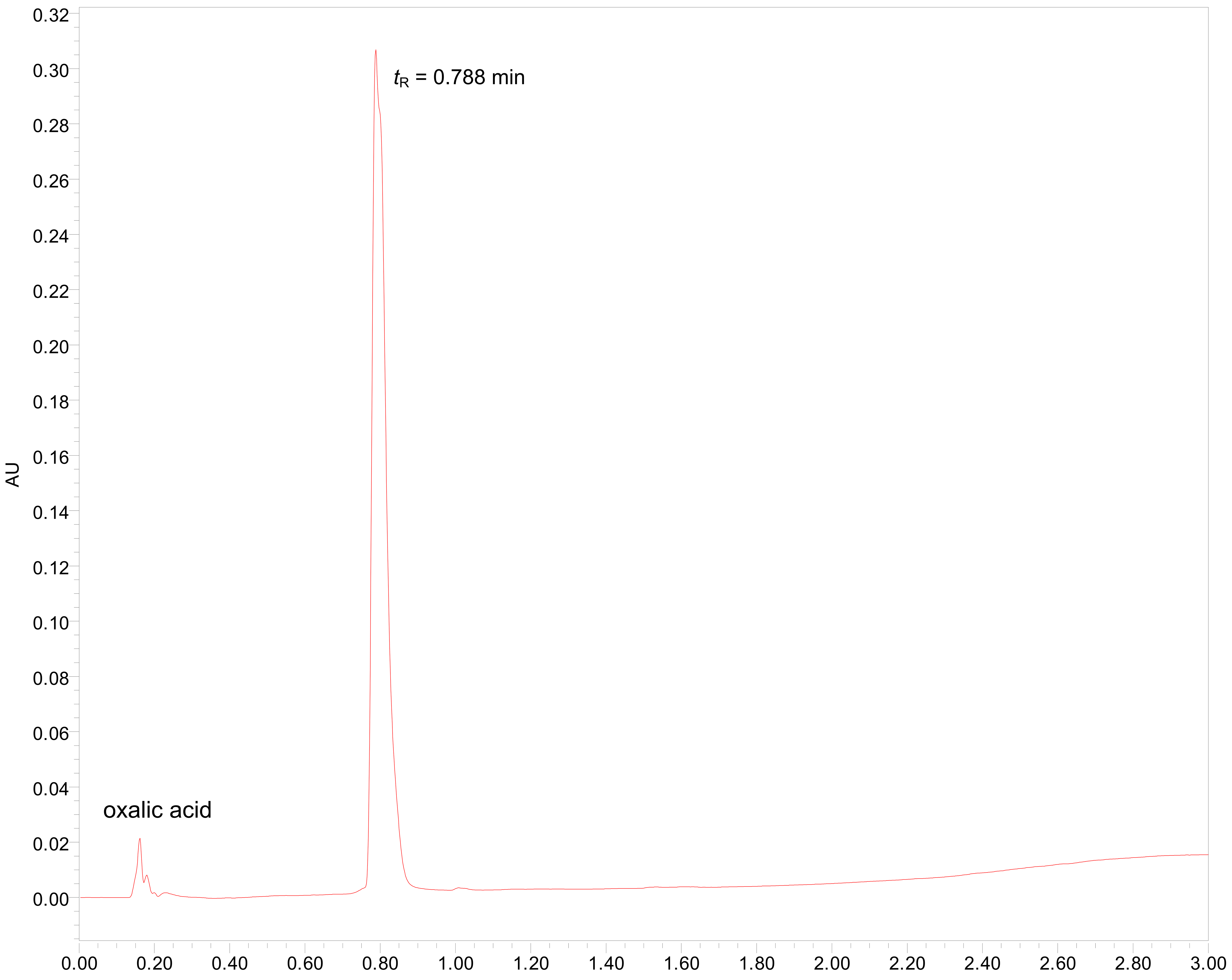

t_R = 0.788 min
oxalic acid
AU
0.32
0.30
0.28
0.26
0.24
0.22
0.20
0.18
0.16
0.14
0.12
0.10
0.08
0.06
0.04
0.02
0.00
0.00
0.20
0.40
0.60
0.80
1.00
1.20
1.40
1.60
1.80
2.00
2.20
2.40
2.60
2.80
3.00

Cpd6

<Chromatogram>

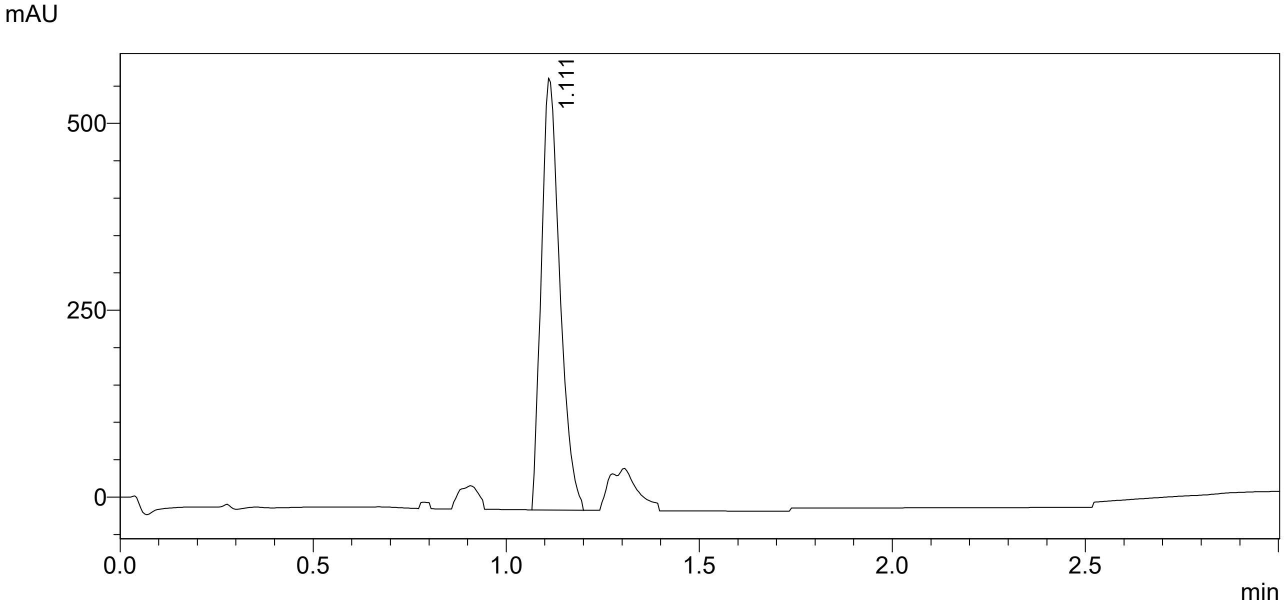

Cpd7

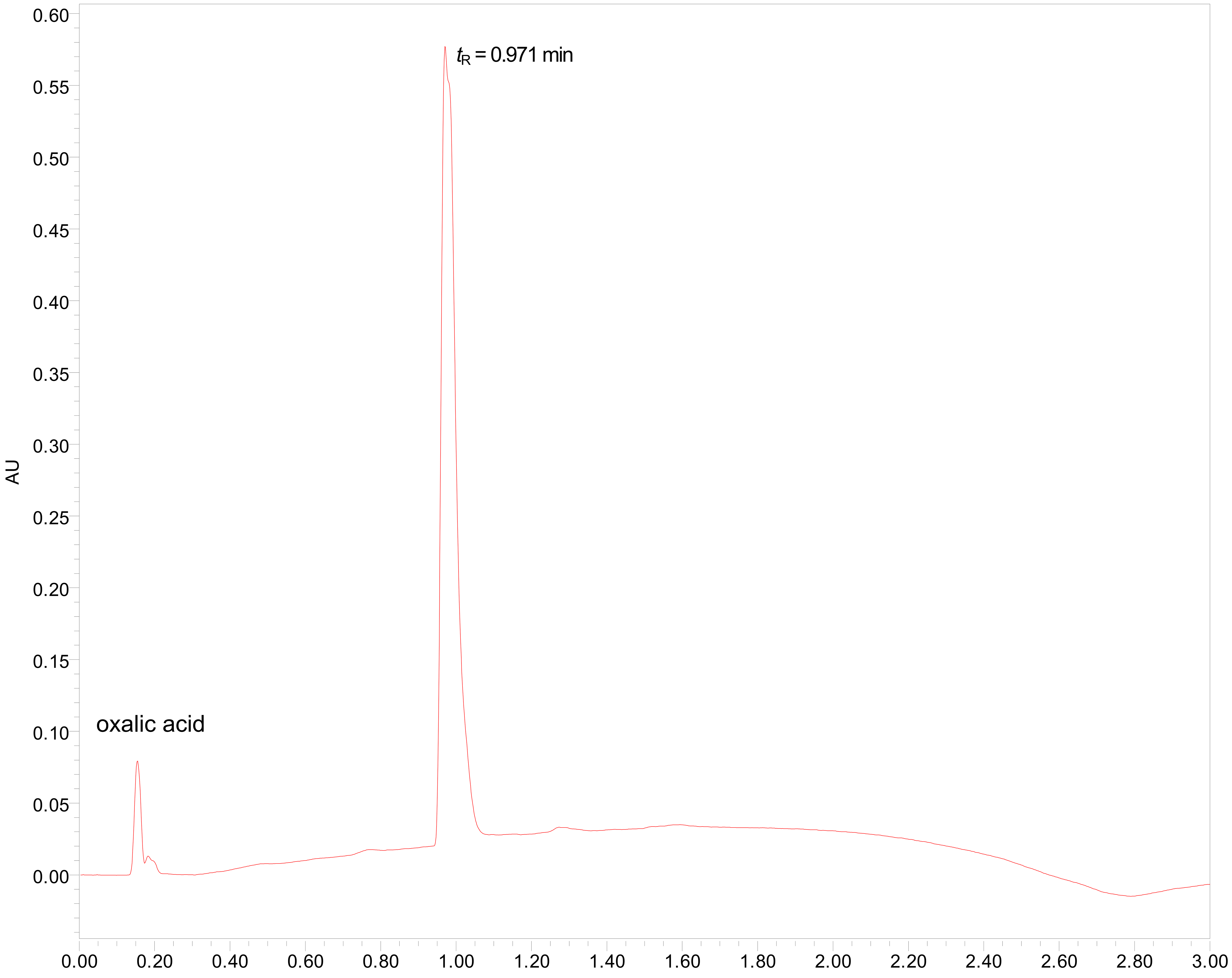

t_R = 0.971 min
oxalic acid
AU
0.60
0.55
0.50
0.45
0.40
0.35
0.30
0.25
0.20
0.15
0.10
0.05
0.00
0.00
0.20
0.40
0.60
0.80
1.00
1.20
1.40
1.60
1.80
2.00
2.20
2.40
2.60
2.80
3.00

SHIMADZU LabSolutions

Cpd8

<Chromatogram>

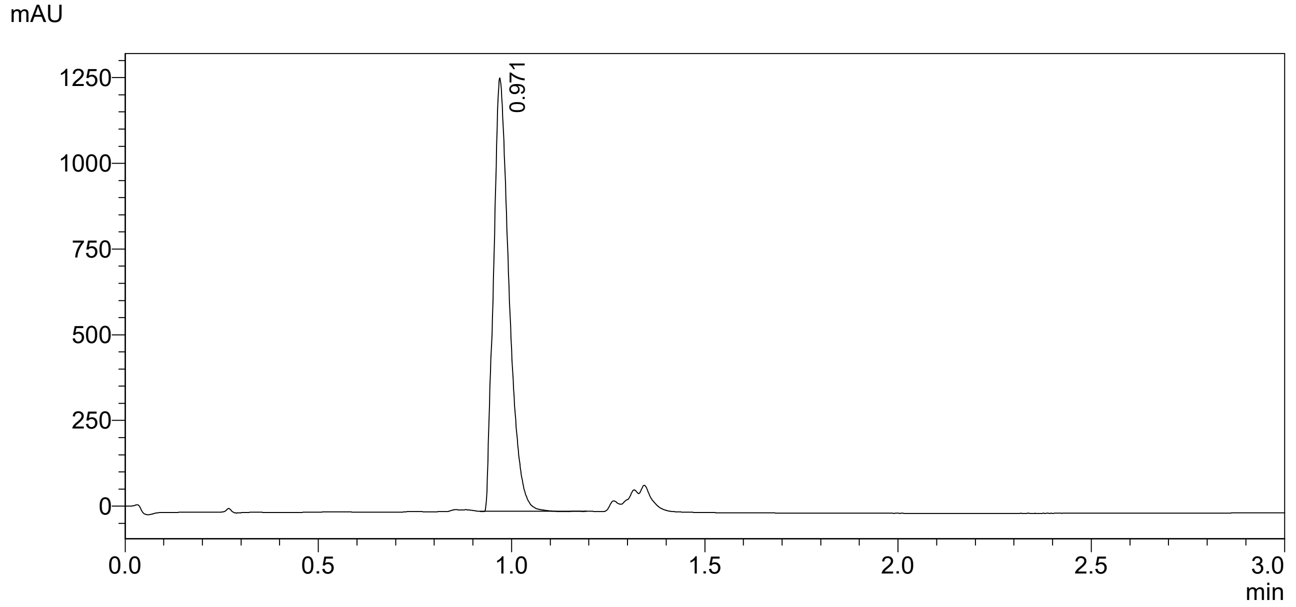

Cpd9

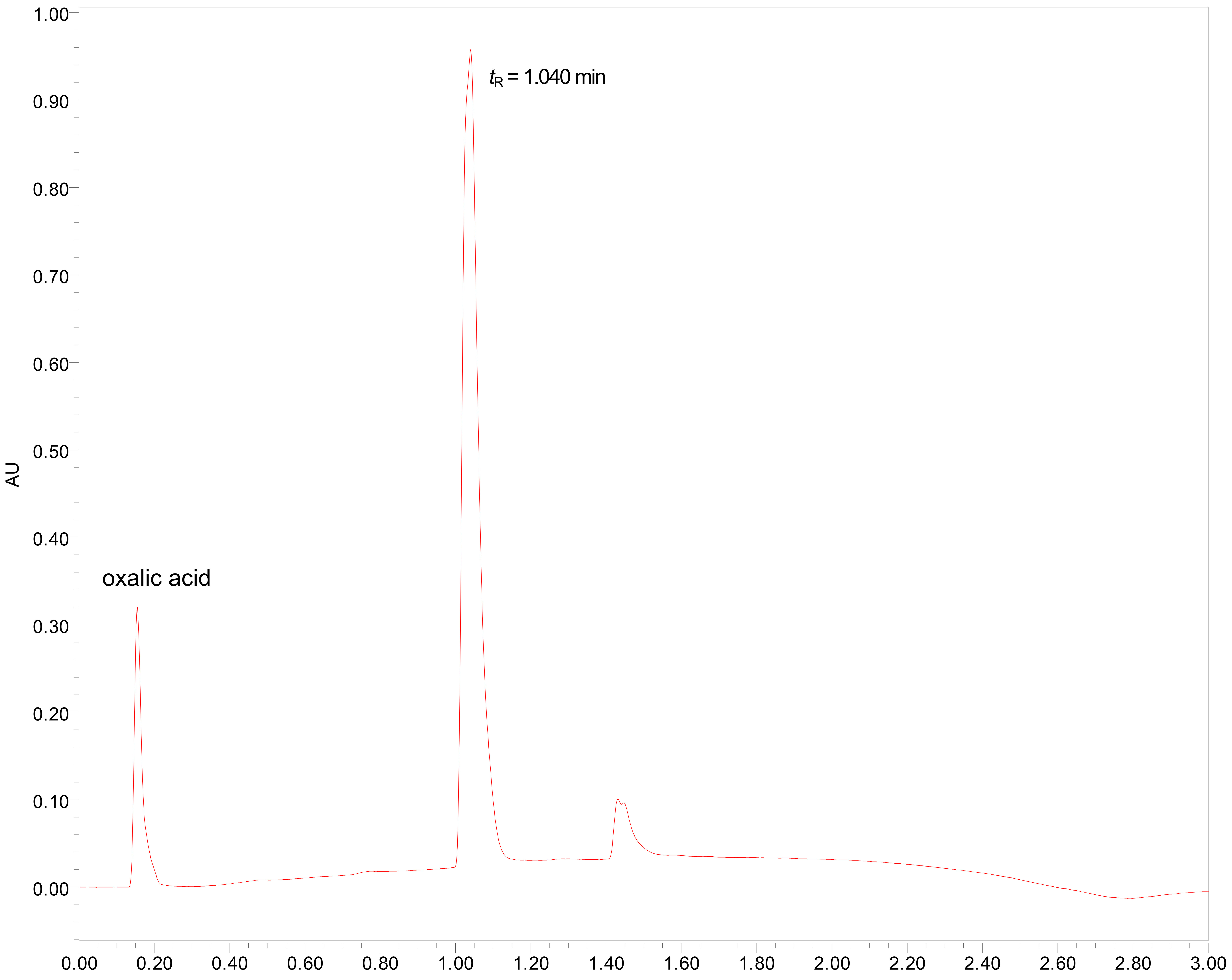

AU
1.00
0.90
0.80
0.70
0.60
0.50
0.40
0.30
0.20
0.10
0.00
t_R = 1.040 min
oxalic acid
0.00
0.20
0.40
0.60
0.80
1.00
1.20
1.40
1.60
1.80
2.00
2.20
2.40
2.60
2.80
3.00

Cpd10

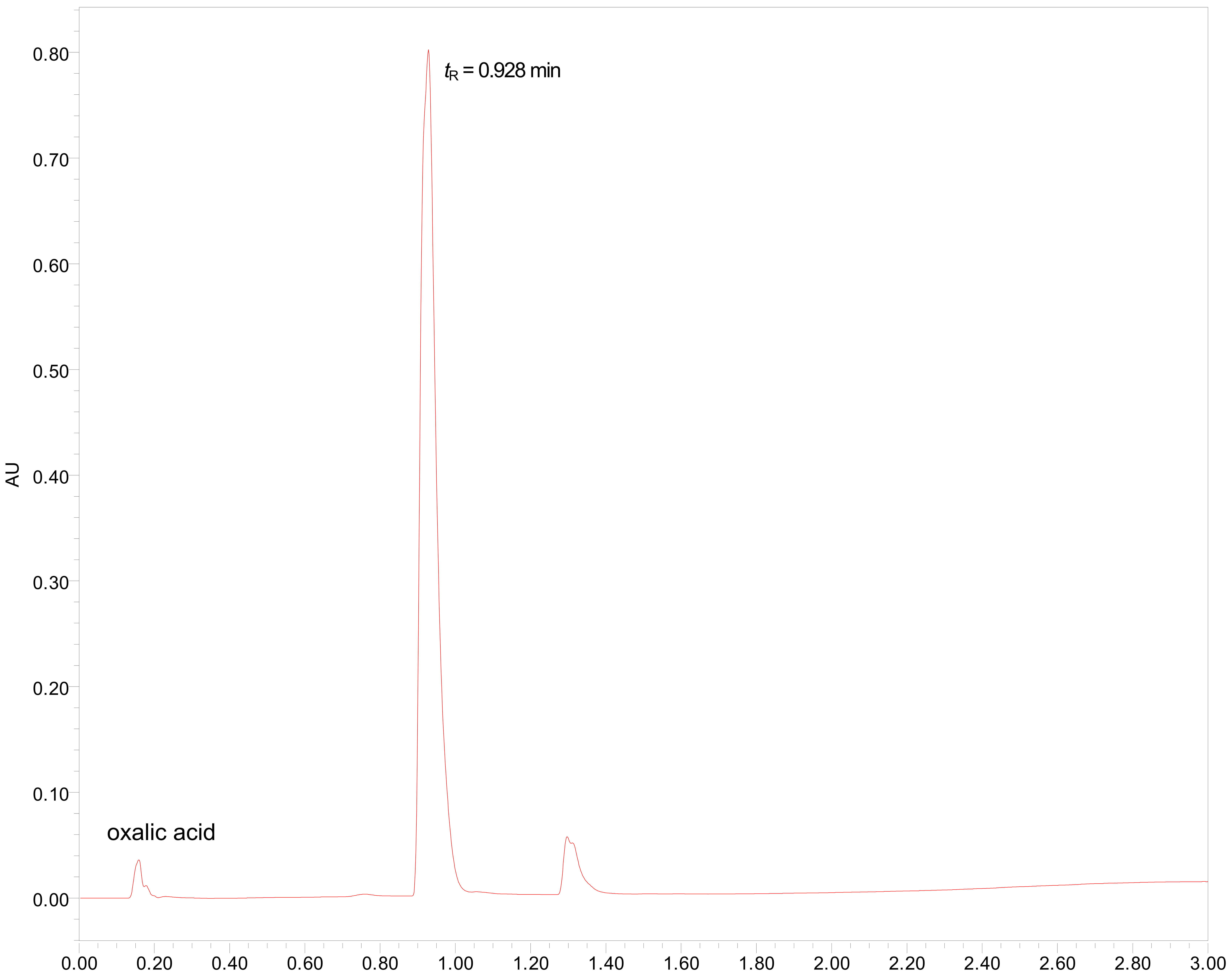

t_R = 0.928 min
oxalic acid
AU
0.00
0.10
0.20
0.30
0.40
0.50
0.60
0.70
0.80
0.00
0.20
0.40
0.60
0.80
1.00
1.20
1.40
1.60
1.80
2.00
2.20
2.40
2.60
2.80
3.00

SHIMADZU LabSolutions

Cpd11

<Chromatogram>

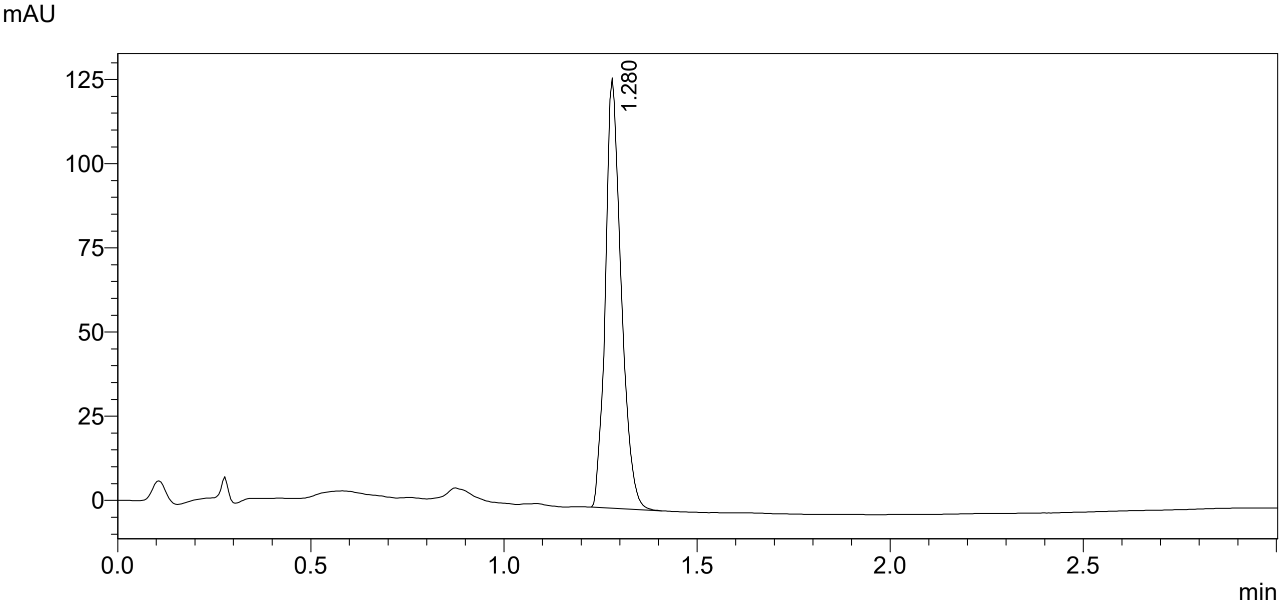

Cpd12

<Chromatogram>

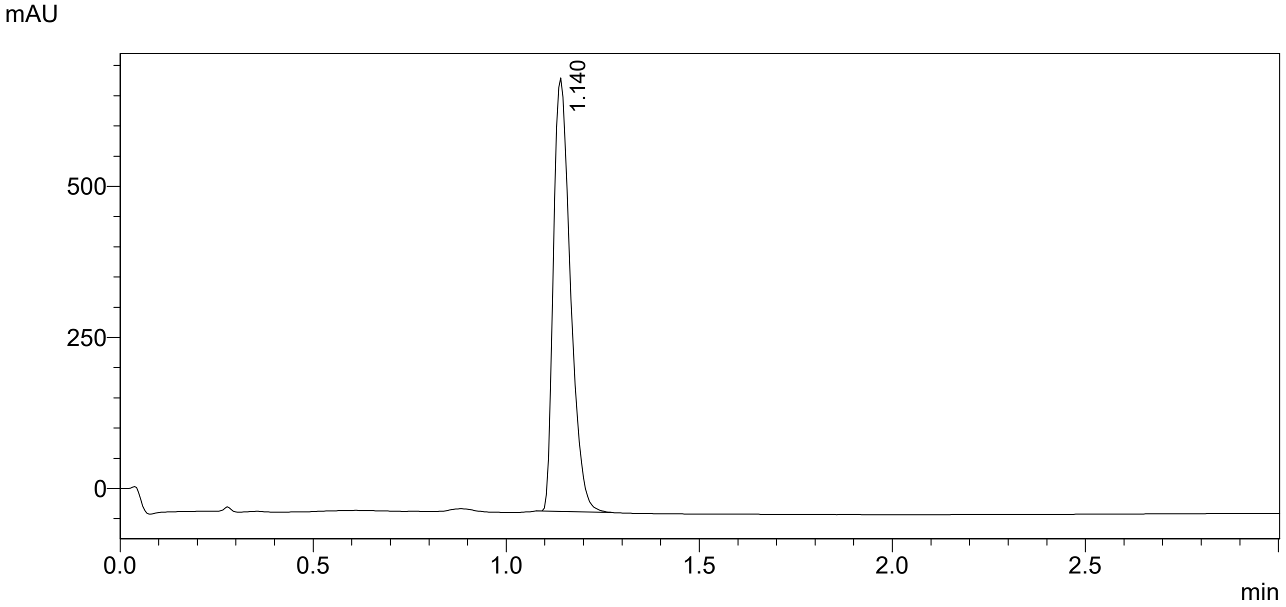

SHIMADZU LabSolutions

Cpd13

<Chromatogram>

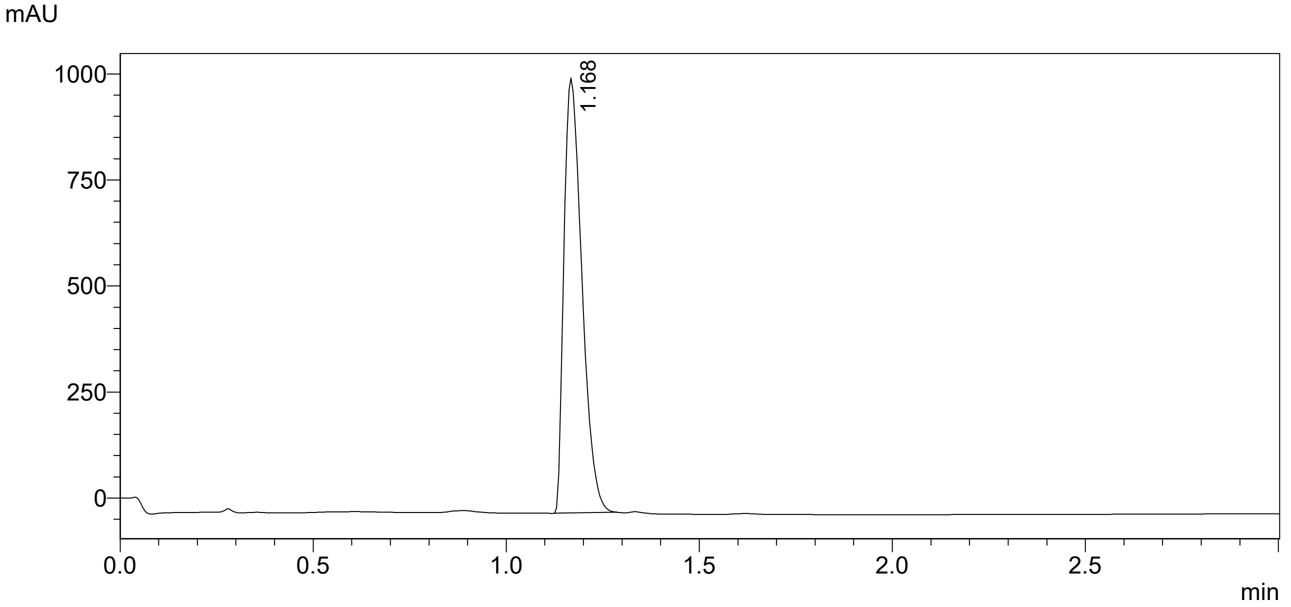

SHIMADZU LabSolutions

Cpd14

<Chromatogram>

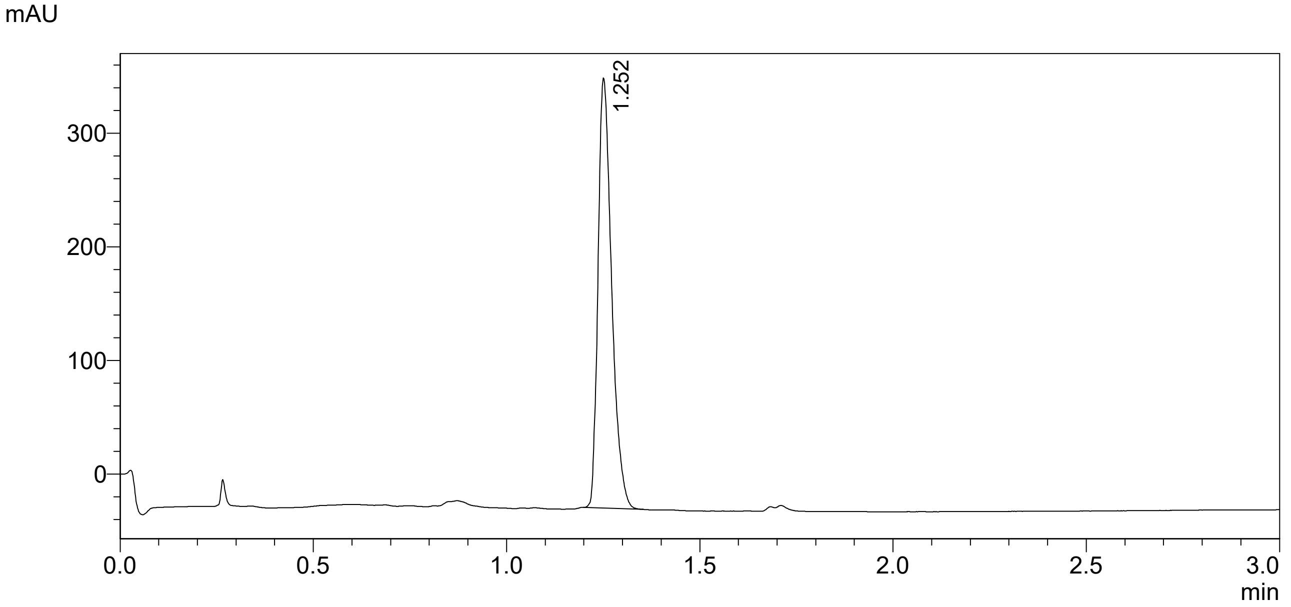

SHIMADZU LabSolutions

Cpd16

<Chromatogram>

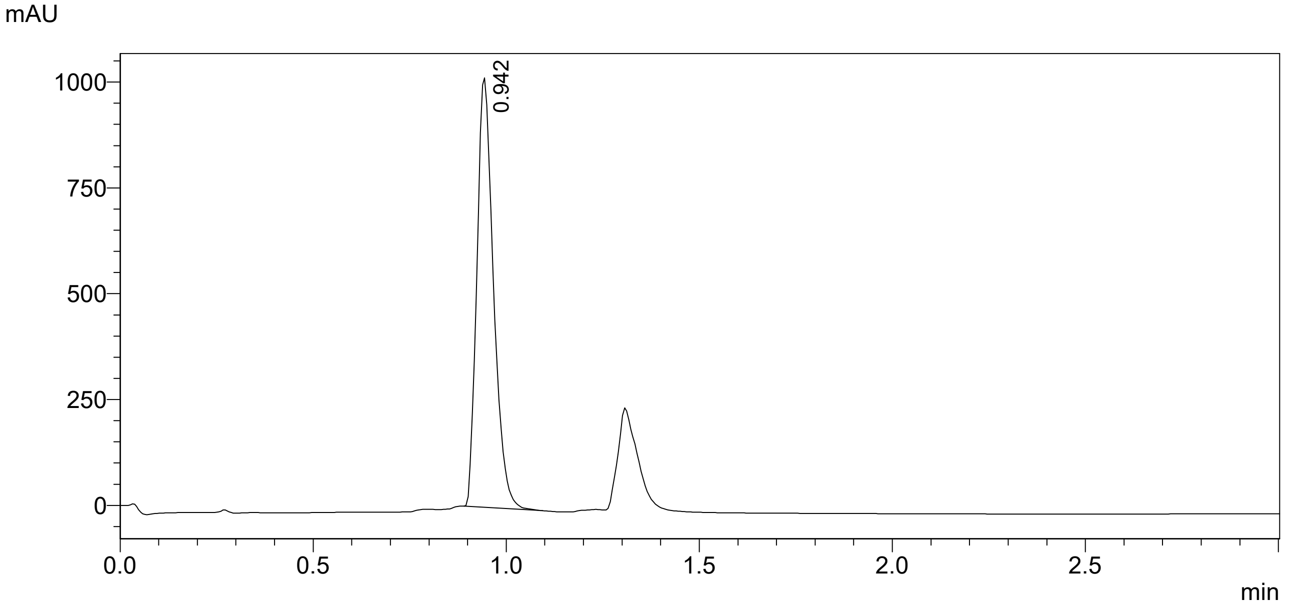

SHIMADZU LabSolutions

Cpd17

<Chromatogram>

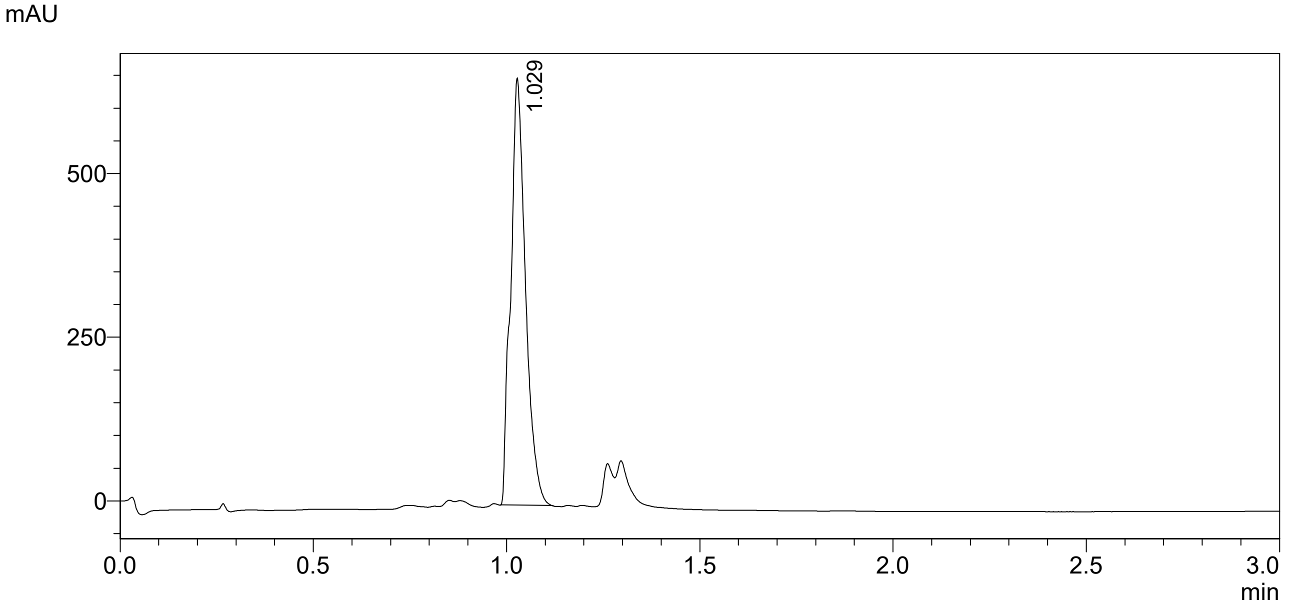

SHIMADZU LabSolutions

Cpd18

<Chromatogram>

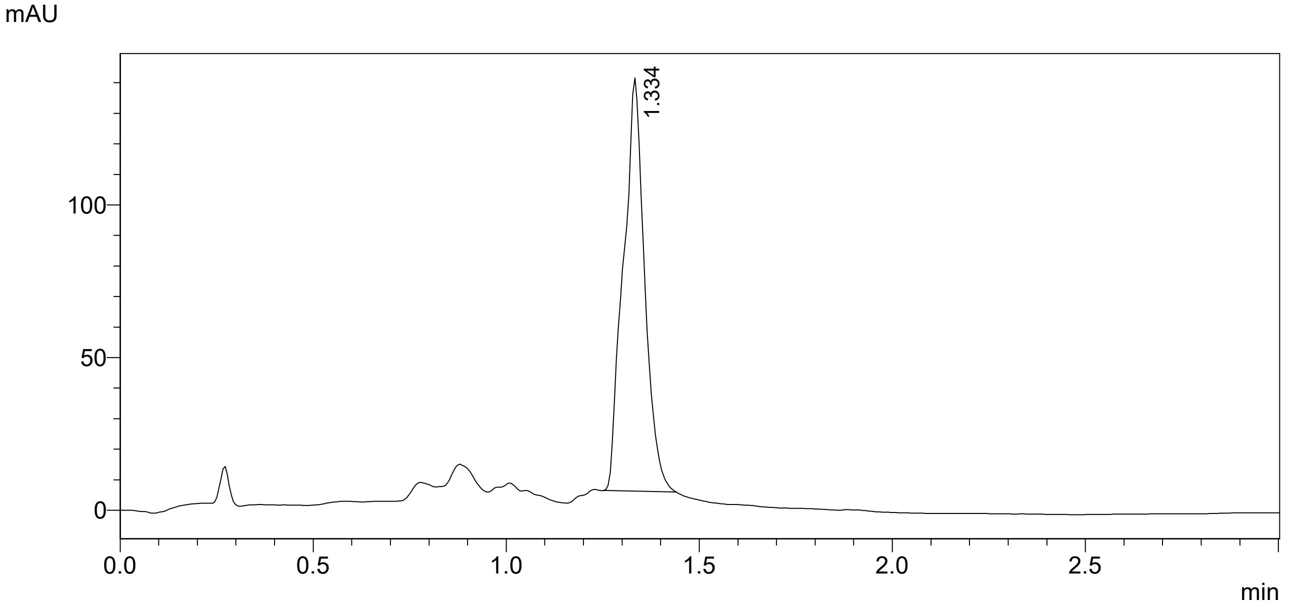

SHIMADZU LabSolutions

Cpd19

<Chromatogram>

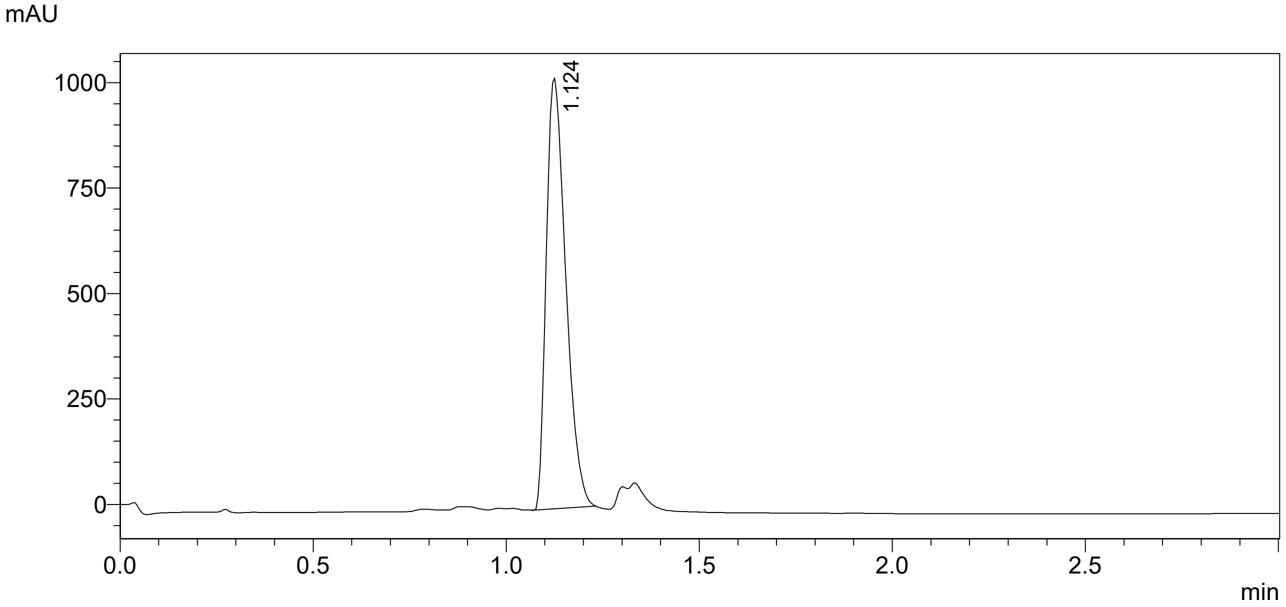

SHIMADZU LabSolutions

Cpd20

<Chromatogram>

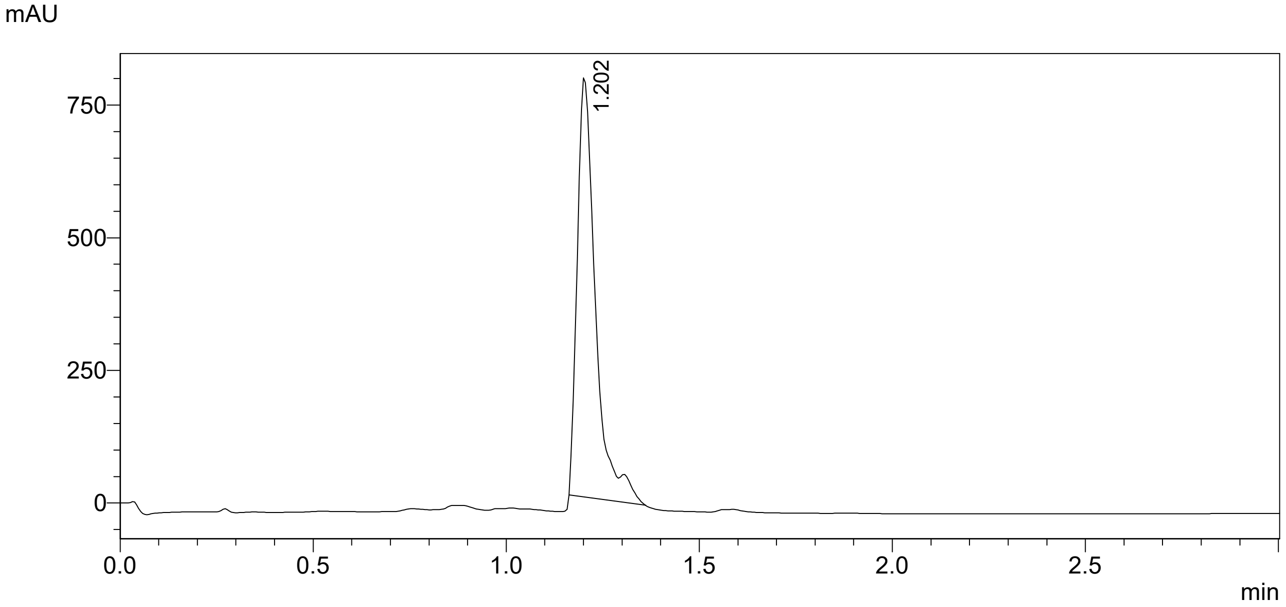

Supplement: Supplementary file 1 — cn1c00435_si_001.pdf [file cn1c00435_si_001.pdf]
